# Dopamine-related alterations in functional brain network dynamic reconfiguration in Parkinson’s disease

**DOI:** 10.1038/s41531-026-01466-w

**Published:** 2026-07-21

**Authors:** AmirHussein Abdolalizadeh, Micha Burkhardt, Paria Jahansa, Carsten Gießing, Karsten Witt, Christiane M. Thiel

**Affiliations:** 1 Biological Psychology Lab, Department of Psychology, School of Medicine and Health Sciences, Carl von Ossietzky Universität Oldenburg, Oldenburg, Germany; 2 Psychological Methods and Statistics, Department of Psychology, School of Medicine and Health Sciences, Carl von Ossietzky Universität Oldenburg, Oldenburg, Germany; 3 Mathematical Psychology, Department of Psychology, School of Medicine and Health Sciences, Carl von Ossietzky Universität Oldenburg, Oldenburg, Germany; 4 Research Center Neurosensory Science, Carl von Ossietzky Universität Oldenburg, Oldenburg, Germany; 5 Department of Neurology, School of Medicine and Health Sciences, Carl von Ossietzky Universität Oldenburg, Oldenburg, Germany

**Keywords:** Diseases, Neurology, Neuroscience

## Abstract

Dopaminergic degeneration in Parkinson’s disease disrupts large-scale brain networks, yet how dopamine loss and its treatment shape the brain’s dynamic reconfiguration over time remains unknown. We combined resting-state fMRI with dopamine transporter scan in 136 drug-naive patients and 20 healthy controls from the PPMI cohort to determine how dopamine transporter availability relates to dynamic network reconfiguration, indexed by how brain regions switch communities over time. Patients showed reduced modular reconfiguration in the default-mode network. Dopamine transporter availability was differentially associated with reconfiguration, showing negative associations in visual and positive associations in limbic networks. Cognitive performance correlated with attention network reconfiguration, whereas motor impairment tracked dopamine loss. Longitudinal analyses in a subset with one year follow-up (*n* = 29) showed that network reconfiguration increased with dopaminergic decline, and medication modulated these dynamics toward the pattern seen in healthy controls. Our findings demonstrate that network reconfiguration captures dopamine-sensitive and cognition-relevant alterations in early Parkinson’s disease.

## Introduction

Parkinson’s disease is the second most common neurodegenerative disorder, clinically characterised not only by motor features such as bradykinesia, rigidity, and resting tremor but also by non-motor symptoms including cognitive impairment, sleep disturbances, olfactory loss, and autonomic dysfunction. The hallmark pathology of Parkinson’s disease is the loss of dopaminergic neurons in the substantia nigra pars compacta^1^, resulting in alterations in task-related and resting-state functional brain networks^2^, with consequences extending beyond motor symptoms, including cognitive impairment^3–6^. While dopamine replacement therapy improves motor symptoms^7^, its impact on cognition and functional networks remains variable and incompletely understood^8^.

Dopamine is critical for cognitive function and exerts differential, often non-linear effects across brain systems, including inverted-U relationships with behavior^9,10^. In healthy individuals, pharmacological manipulations of the dopaminergic system reveal network-specific associations, with dopamine administration enhancing some networks while diminishing others^11,12^. In Parkinson’s disease, progressive dopaminergic loss further complicates this relationship by altering baseline levels and modulating dynamics across brain systems and behavioral domains^13^. Given that optimal dopamine levels further differ between cognitive processes^14,15^, variability in treatment effects may reflect domain- and network-specific neuromodulation.

Functional connectivity is not static but fluctuates over time, and dynamic functional connectivity (dFC) has emerged as a powerful tool for capturing these time-varying co-fluctuations in brain activity^16,17^. Time-resolved or sliding window analyses estimate connectivity in short, overlapping segments of the functional MRI time series, to capture how functional connections evolve. Beyond simply describing dFC, the time-resolved matrices can be treated as layers in a multilayer network. This allows community detection algorithms to identify how brain regions shift their modular allegiance over time, a property known as dynamic network reconfiguration^18–20^. Dynamic network reconfiguration provides a mechanistically meaningful marker of how flexibly large-scale brain systems adapt over time. By applying this method, altered dFC reconfiguration measures have been reported across neurological and psychiatric disorders, such as schizophrenia^21^, post-traumatic stress disorder^22^, Alzheimer’s disease^23^, multiple sclerosis^24^, and Lewy body dementia^25^. In Parkinson’s disease, multilayer community detection has been applied mainly in medicated later-stage patients, showing higher reconfiguration frequency OFF relative to ON medication^26^. Yet, early stages of the disease, where network reconfiguration may offer a potentially sensitive marker of emerging dopaminergic dysfunction and early clinical changes remain unexplored, leaving it unclear how these dynamics relate to nigrostriatal dopaminergic integrity at baseline and how they evolve with disease progression and initiation of dopaminergic therapy.

Here, we combine dopamine transporter scan (DaTscan) derived striatal dopamine transporter (DaT) availability, as a measure of nigrostriatal dopaminergic integrity, with resting-state fMRI (rsfMRI) in early, drug-naive Parkinson’s disease and healthy controls. We quantify subject-level network reconfiguration measures, namely flexibility, promiscuity, cohesion, and disjointedness, via multilayer community detection at nodal, network, and whole-brain levels. We test associations with cognition and motor impairment. We further evaluate longitudinal change over one year in a small subset of patients that had one-year follow-up imaging, including the modulatory effects of initiating dopaminergic medication. We hypothesized that early Parkinson’s disease is characterised by network-specific alterations in dynamic reconfiguration, that these alterations relate to DaT availability and cognitive performance, and that initiating dopaminergic therapy modulates these dynamics over time.

## Results

### Participant demographics and clinical characteristics

The final sample comprised 136 patients with Parkinson’s disease and 20 healthy controls. The groups did not differ significantly in age, sex, or MoCA scores (all *p* > 0.05; see Table 1). The patients showed the expected motor impairment with significantly higher MDS-UPDRS Part 3 scores and reduced dopamine transporter binding across all subcortical regions.

**Table 1:** Group differences in demographics, striatal dopamine transporter availability, and dynamic reconfiguration measures

	Healthy Controls (*n* = 20)	Parkinson’s Disease (*n* = 136)	*FDR- Corrected p-value*
**Demographics and Clinical Data**			
Sex = M (%)	11 (55.0)	92 (67.6)	0.451
Age	62.30 (12.93)	64.11 (9.50)	0.451
Disease Duration (Days)	—	290.43 (288.53)	
MDS-UPDRS Part 3 Total Score^†^	0.00 [0.00, 2.00]	21.00 [15.00, 31.00]	< 0.001*
Hoehn & Yahr Stage^†^	0.00 [0.00, 0.00]	2.00 [1.00, 2.00]	< 0.001*
Total MoCA Score^†^	28.00 [26.75, 29.25]	27.00 [26.00, 29.00]	0.16
**DaTscan striatal binding ratios**			
Right Caudate	3.38 (0.56)	2.11 (0.65)	< 0.001*
Left Caudate^†^	3.40 [3.02, 3.71]	2.06 [1.64, 2.47]	< 0.001*
Right Putamen^†^	2.47 [2.12, 2.74]	0.86 [0.70, 1.17]	< 0.001*
Left Putamen^†^	2.41 [2.18, 2.70]	0.84 [0.66, 1.13]	<0.001*
Striatum Average^†^	2.76 [2.66, 3.20]	1.47 [1.20, 1.75]	<0.001*
**Dynamic Reconfiguration Measures (Whole-brain average)**^**a**^			
Flexibility	0.16 (0.01)	0.15 (0.01)	0.249
Promiscuity	0.56 (0.03)	0.56 (0.02)	0.870
Disjointedness	2.42e-03 (1.90e-04)	2.35e-03 (1.76e-04)	0.103
Cohesion	0.16 (0.01)	0.15 (0.01)	0.258

^a^Reconfiguration measures are derived from the sliding window length of 19 TRs, with the brain parcellated and segmented based on Schaefer’s cortical and Melbourne’s subcortical atlas.

Variables with † had non-normal distribution, thus they are reported as median [Interquartile range] and were tested with non-parametric tests. (*) marks the significant differences after correcting for multiple comparisons. The Benjamini-Hochberg false discovery rate method was used to correct for multiple comparisons.

### Alterations in functional brain network reconfiguration in Parkinson’s disease

Reconfiguration measures were calculated at the nodal, network-average, and whole-brain average levels. Nodal measures were derived using the Schaefer cortical atlas^27^ together with the Melbourne subcortical atlas^28^. These reconfiguration measures were computed over three successive time windows of different lengths within a range previously suggested for dynamic connectivity analysis^17^. These sliding windows (SW) were defined based on the numbers of repetition time (TR = 2.5 s) and are referred to as SW15, SW19, and SW23, corresponding to window lengths of 37.5 s, 47.5 s, and 57.5 s, respectively. Network-level measures were obtained by averaging nodal reconfiguration values across regions belonging to the predefined functional networks of the Schaefer parcellation. Subcortical regions were averaged into a single SUB network. Whole-brain average network reconfiguration measures were calculated by averaging the metrics across all nodes. To assess spatial robustness, cortical nodal reconfiguration measures were additionally computed using the Glasser multi-modal parcellation^29^. Due to differences in spatial granularity and regional boundaries between parcellations, direct node-to-node correspondence is not feasible. However, whole-brain average reconfiguration measures showed strong cross-parcellation correspondence across all sliding window lengths and metrics (*p* < 0.001). Flexibility and cohesion demonstrated the highest agreement (both *r* = 0.82), followed by disjointedness (*r* = 0.59–0.64), while promiscuity showed comparatively weak agreement (*r* = 0.22–0.31).

**Fig. 1:**
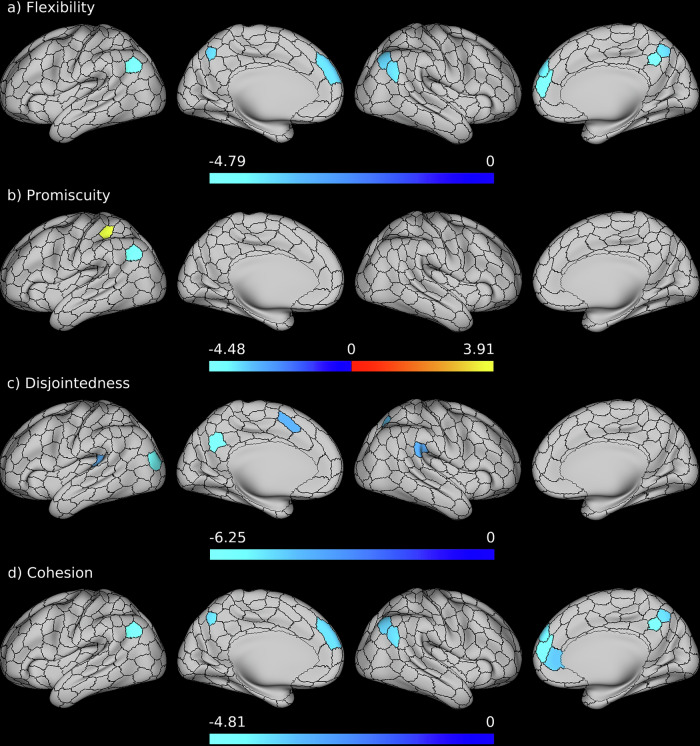
Nodes showing significant group differences in reconfiguration measures between Parkinson’s disease patients and healthy controls. Displayed results show group differences in nodal (**a**) flexibility, (**b**) promiscuity, (**c**) disjointedness, and (**d**) cohesion. The reconfiguration measures are based on the SW19 window length and the Schaefer cortical parcellation. Colorbars show t-values; regions marked in blue and yellow indicate significantly lower and higher values (FDR corrected) in patients compared to healthy controls, respectively. No significant differences were found for subcortical regions.

A first analysis examined group differences in reconfiguration measures at whole-brain, network, and nodal levels. Whole-brain average reconfiguration measures did not differ between groups (Table 1; Supplementary Table 1). However, at the network level, patients showed lower flexibility, promiscuity, and cohesion in the default-mode network (DMN) than controls across all sliding window lengths tested (all corrected *p*-values < 0.05). For example, in SW19, the DMN flexibility was lower in patients compared with controls (mean (SD): 0.143 (0.016) vs. 0.151 (0.016); Cohen’s *d* = −0.50 [95% CI: –0.77, –0.22]; Suppl. Table 2). At the nodal level, patients showed reduced flexibility, cohesion, promiscuity, and disjointedness, primarily in the frontal and parietal areas, overlapping largely with regions associated with the DMN (Fig. 1, Supplementary Figures 1 and 2). Importantly, similar spatial patterns were observed when the analysis was repeated using the Glasser atlas, despite the differences in parcellation schemes between the two atlases. Although the exact parcel definitions differ, the regions showing significant group differences were spatially mapped to cortical areas corresponding to the nodes of DMN, supporting the robustness of group differences across parcellation approaches (Suppl. Figs. 3–5).

### Network-specific associations between dopamine and functional brain network reconfiguration

Next, we investigated how striatal DaT availability, as measured by dopamine transporter striatal binding ratio (DaTscan SBRs), relates to reconfiguration measures at whole-brain, network, and nodal levels in patients and healthy controls. We included averaged DaTscan values for the bilateral caudate (caudate average), bilateral putamen (putamen average), and their combined average (striatum average) as an overall index of striatal DaT availability, and entered each measure separately into the analyses while adjusting for age and sex. No significant relationships between DaT availability and whole-brain average reconfiguration measures were observed in the patients. Within healthy controls, whole-brain average flexibility and cohesion were significantly associated with caudate average DaT availability (Estimate (Std. Error) = 0.011 (0.004), corrected *p* = 0.022, for both measures). Whole-brain average promiscuity was also associated with putamen average (Estimate (Std. Error) = 0.062 (0.012), corrected *p* = 0.001) and the striatum average DaT availability (Estimate (Std. Error) = 0.056 (0.012), corrected *p* = 0.002).

**Fig. 2:**
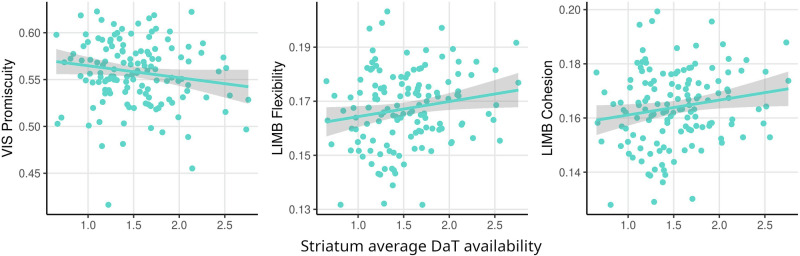
Significant associations between reconfiguration measures on the network level and striatum average DaT availability in Parkinson’s disease patients. Reconfiguration measures of visual and limbic networks show different associations with striatum average DaT availability, with the promiscuity of the visual network showing a significant negative association and the flexibility and cohesion of the limbic network revealing significant positive associations. Reconfiguration measures are derived from the sliding window length of 19 TRs, with the brain parcellated and segmented based on Schaefer’s cortical and Melbourne’s subcortical atlas. DaT Dopamine Transporter, VIS Visual network, LIMB Limbic network.

At the network level, DaT availability in the patients exhibited distinct associations with reconfiguration measures across specific networks (Fig. 2). The striatum average was negatively associated with visual network (VIS) promiscuity (Estimate (Std. Error) = −0.021 (0.006), corrected *p* = 0.025), and positively with limbic network (LIMB) flexibility and cohesion (both with Estimate (Std. Error) = 0.007 (0.002), corrected *p* = 0.029). In healthy controls, DaT availability was positively associated with multiple network-level reconfiguration metrics, including flexibility of somatomotor (SM), salience-ventral and dorsal attention networks (SAL, DAN), LIMB, DMN; promiscuity of the control (CONT) and subcortical (SUB) networks, and cohesion of the SM, SAL, and DMN networks (all corrected *p* < 0.05, Supplementary Table 3). In other words, we found that DaT availability in Parkinson’s disease patients was associated with only a small number of networks and showed mixed, network-specific relationships, whereas healthy controls exhibited widespread, exclusively positive associations across multiple networks.

At the nodal level, a similar pattern emerged in both groups. In patients, DaT availability was associated with reconfiguration measures of LIMB and VIS network nodes, but these associations did not survive multiple-comparison correction (Suppl. Table 4). In healthy controls, in contrast, a broad set of nodal reconfiguration measures across SM, SAL, DAN, CONT, DMN, VIS, and SUB networks showed significant positive associations with DaT availability (Suppl. Figs. 6–8). To assess robustness across parcellations, we repeated the nodal analysis in patients with Parkinson’s disease using the Glasser atlas. This analysis yielded a small number of significant associations with striatum average DaT availability, including a positive association with right hippocampal promiscuity and a negative association with a region in the right insula (right insular granular, Ig; Suppl. Table 5). Importantly, the regions identified using the Glasser atlas correspond anatomically to areas showing strong uncorrected associations in the Schaefer-based analysis, indicating a largely consistent spatial pattern across parcellations despite differences in parcel definitions (Supplementary Fig. 9, shown for Parkinson’s disease patients).

### Divergent roles of network reconfiguration and dopamine in cognitive and motor performance in Parkinson’s disease

In a further step, we investigated how reconfiguration measures relate to cognitive and motor performance within the patient group, adjusting for age and sex and in addition education for cognitive performance. Our analysis revealed that reconfiguration measures in patients were positively associated with MoCA scores, which we used as a proxy for global cognitive performance. At the whole-brain level, higher MoCA scores were associated with increased flexibility (Estimate (Std. Error): 32.5 (13.6), corrected *p* = 0.038) and cohesion (Estimate (Std. Error): 32.7 (13.7), corrected *p* = 0.037) only in the SW15 window. Similar effect directions were observed for the other window lengths, although these did not survive multiple-comparison correction.

At the network level, MoCA scores in the SW15 window were positively associated with flexibility (Estimate (Std. Error) = 36 (12), corrected *p* = 0.017) and cohesion (Estimate (Std. Error) = 36 (12), corrected *p* = 0.014) of the dorsal attention network (DAN). For SW19, these associations approached the corrected significance threshold (flexibility: Estimate (Std. Error) = 33 (12), corrected *p* = 0.056; cohesion: Estimate (Std. Error) = 33 (12), corrected *p* = 0.057), while they did not reach significance after correction in the longer SW23 window (flexibility: Estimate (Std. Error) = 31 (12), corrected *p* = 0.095; cohesion: Estimate (Std. Error) = 31 (12), corrected *p* = 0.093; Suppl. Table 6). Motor performance, as measured by MDS-UPDRS Part 3 scores, showed no significant associations with network-based reconfiguration measures across all sliding window lengths.

**Fig. 3:**
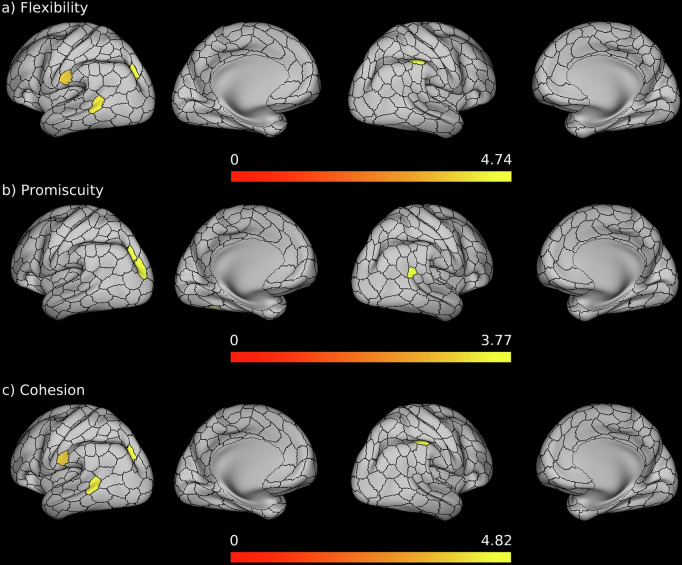
Significant associations between clinical scores and nodal reconfiguration measures in Parkinson’s disease patients. Displayed results show significant nodal (**a**) flexibility, (**b**) promiscuity, and (**c**) cohesion associations. The reconfiguration measures are based on the SW19 window length and the Schaefer cortical parcellation. Colorbars show t-values; regions marked in yellow–red indicate positive associations between nodal reconfiguration measures and MoCA scores. Higher values across nodes in the dorsal attention and control networks were associated with better cognitive performance. No significant associations were observed for subcortical regions.

At the nodal level, a similar pattern emerged, with positive associations between MoCA scores and nodes in the DAN and CONT (Fig. 3, Suppl. Figs. 10, 11). Using Glasser’s parcellation, fewer but anatomically similar regions, particularly for flexibility and cohesion, including the superior temporal visual area (STV), were positively associated with MoCA in the SW15 and SW23 windows (Suppl. Figs. 12, 13). With respect to motor performance, the nodal results were less consistent across sliding windows and parcellations. In Schaefer’s parcellation, a small number of regions in the SW15 window showed significant associations after multiple-comparison correction (Suppl. Fig. 14), whereas no significant findings were observed in SW19 or SW23. Using Glasser’s parcellation, negative associations between flexibility and motor symptom severity were identified in areas 47S, anterior agranular insular complex (AAIC), and superior temporal sulcus - dorsal posterior (STSdp) across all sliding window lengths (Suppl. Figs. 15–17). However, these regions did not directly correspond to those identified using the Schaefer atlas.

In contrast to the MoCA-related findings, which showed consistent involvement of DAN and CONT networks across multiple windows and parcellations, the motor-related nodal results were more variable and did not demonstrate a similarly robust or spatially consistent pattern (Supplementary Fig. 9).

As expected, in patients, all striatal DaT availability measures were negatively associated with motor performance (all corrected *p* < 0.05, Estimate (Std. Error) for DaT availability as in striatum average: −4.61 (1.66), for putamen average: −6.32 (2.18), and for caudate average: −3.01 (1.22)). However, we found no evidence of a relationship between DaT availability and cognitive performance (all corrected *p* > 0.05, Estimate (Std. Error) for striatum average: −0.072 (0.383), for putamen average: −0.700 (0.502), and for caudate average: 0.124 (0.284)). Together, these findings suggest that while DaT availability is primarily linked to motor impairment, dynamic reconfiguration measures are more closely associated with cognitive performance in Parkinson’s disease.

### Flexibility of dorsal attention and control networks relates to executive function performance

Given the significant network-average and nodal flexibility associations of DAN and CONT with MoCA, which provides a general measure of cognitive function, we conducted an additional post-hoc analysis to examine whether these dynamic reconfiguration metrics were also related to domain-specific cognitive measures. To this end, we included Trail Making Test (TMT) scores, specifically TMT-A and TMT-B minus TMT-A (TMTB-A). TMT-A primarily reflects processing speed and visual attention, whereas TMTB-A is commonly used as a more specific measure of executive function. These tests were selected because attentional control and executive function are commonly affected in Parkinson’s disease^30^, and are also closely supported by DAN and CONT^31^.

After adjusting for age, sex, and education, we observed a significant negative association between DAN flexibility and TMTB-A scores in all sliding window lengths tested (For SW15: Estimate (Std. Error) = −307.99 (137.29), *p* = 0.026; SW19: Estimate (Std. Error) = −283.89 (133.66), *p* = 0.035; SW23: Estimate (Std. Error) = −279.89 (132.34), *p* = 0.036). Flexibility of the CONT network showed a similar pattern, reaching significance in SW15 (Estimate (Std. Error) = −321.11 (124.63), *p* = 0.011) and approaching significance in SW19 (Estimate (Std. Error) = −241.87 (122.87), *p* = 0.051) and SW23 (Estimate (Std. Error) = −236.46 (121.29), *p* = 0.053). No significant associations were observed for TMT-A (all *p* > 0.05). Because lower TMTB-A values indicate better set-shifting and executive function, these findings suggest that greater flexibility of attention- and control-related networks is associated with better executive performance in Parkinson’s disease.

### Progressive dopamine loss amplifies alterations in functional brain network reconfiguration over time

**Fig. 4:**
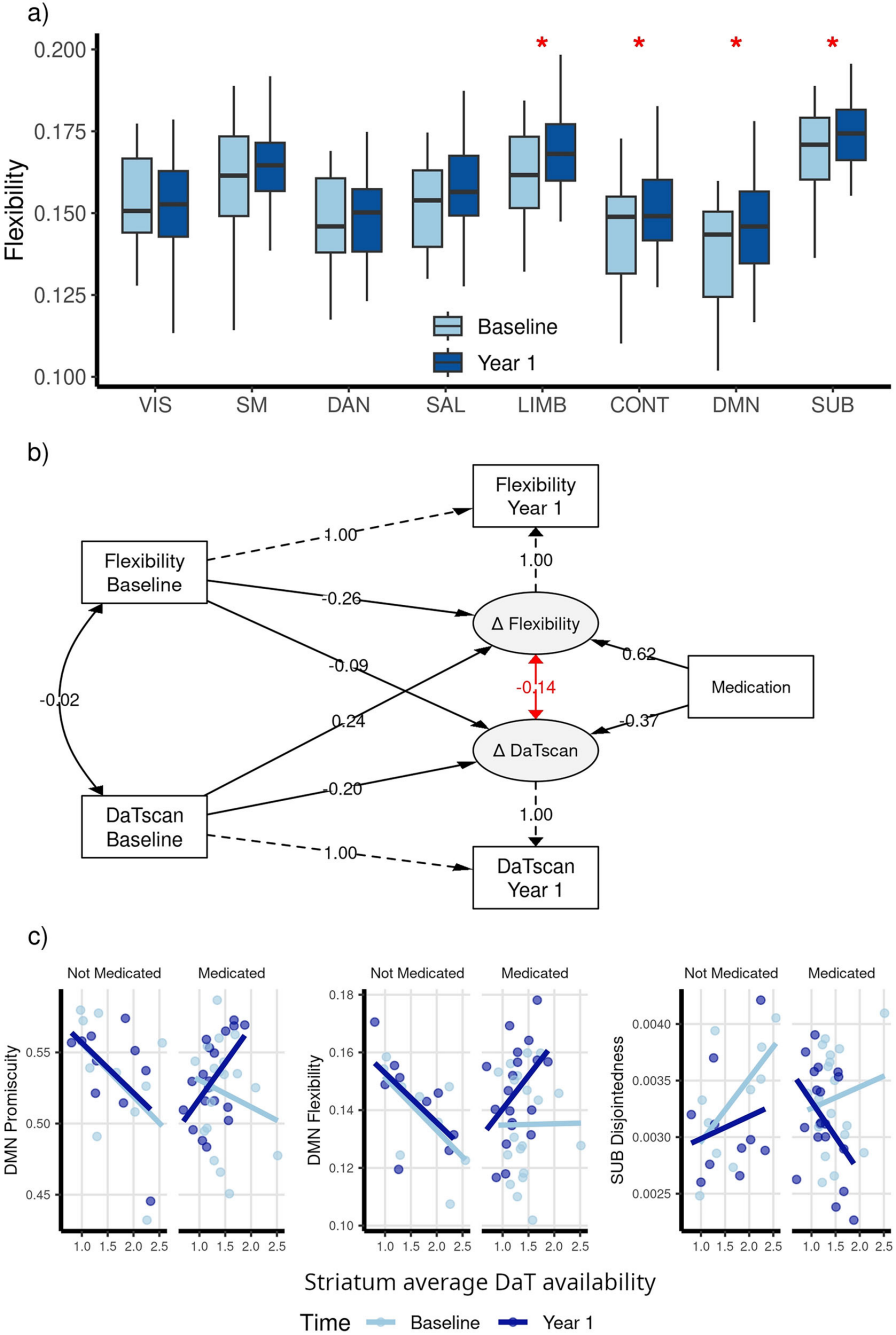
Longitudinal changes in network reconfiguration, their relationship with striatal DaT availability, and the effects of medication in Parkinson’s disease patients. **a** Flexibility was increased in several networks from baseline (light blue) to year 1 (dark blue; asterisks indicate significant results with FDR-corrected *p* < 0.05). **b** Example path diagram of the bivariate latent change score model with baseline and follow-up measures of whole-brain average flexibility and the striatum average measure of DaT availability (DaTscan baseline and Year 1). The model revealed a significant negative covariance between the two latent change variables from baseline to year one (red arrow). Significance was assessed using 95% posterior credible intervals. Values on the paths represent posterior mean parameter estimates from the model. The correlation between latent change factors (ρ) is not shown in the figure but is derived from their covariances and variances and reported in the main text. Panel (**c**) illustrates that in unmedicated patients, the association between striatum average DaT availability and network reconfiguration remained similar from baseline (light blue line and circle) to follow-up (dark blue line and circle). In contrast, once patients started dopaminergic medication, these associations changed markedly. Specifically, medication reversed the relationship between striatal DaT availability and reconfiguration measures for DMN promiscuity (left), DMN flexibility (middle), and disjointedness in the subcortical network (right). Reconfiguration measures are derived from the sliding window length of 19 TRs, with the brain parcellated based on Schaefer’s cortical and Melbourne’s subcortical atlas. DaT Dopamine transporter, Networks are abbreviated as VIS visual, CONT control, DMN Default-mode, DAN Dorsal attention, LIMB Limbic, SAL Ventral attention-salience, SM Somatomotor, SUB Subcorticals.

A final analysis focused on the subset of patients with one-year follow-up data (n = 29), to examine longitudinal changes in network reconfiguration and their relationship with striatal DaT availability and medication status. Over one year of disease progression, whole-brain flexibility and cohesion increased (Cohen’s *d* [95% CI] = 0.48 [0.26, 0.70] and 0.49 [0.27, 0.71]), while disjointedness and DaT availability values declined (For disjointedness: Cohen’s *d* [95% CI] = −0.43 [−0.64, −0.21]; Table 2). Consistent with these global changes, flexibility and cohesion increased on the network level in LIMB, CONT, DMN, and SUB (Fig. 4a). At the nodal level, no changes survived multiple comparison corrections. These findings were robust across sliding window lengths (Suppl. Tables 7 and 8).

**Table 2:** Changes in clinical scores, striatal dopamine transporter availability, and dynamic reconfiguration measures in Parkinson’s disease patients (*n* = 29) after one year

	Baseline	Year one	FDR- Corrected *p*-value
**Clinical Data**			
MDS-UPDRS Part 3 Total Score	21.76 (9.22)	26.96 (10.18)	0.132
Total MoCA Score	27.59 (2.34)	27.21 (2.15)	0.349
Medicated	0 (0%)	19 (65.5%)	
Hoehn & Yahr stage	1.71 (0.46)	1.61 (0.56)	0.349
**DaTscan striatal binding ratios**			
Right Caudate^†^	2.01 [1.63, 2.42]	1.94 [1.54, 2.17]	0.057
Left Caudate	2.15 (0.71)	1.86 (0.57)	0.003*
Right Putamen	0.89 (0.33)	0.80 (0.33)	0.011*
Left Putamen	0.88 (0.37)	0.83 (0.33)	0.239
Striatum average	1.52 (0.45)	1.35 (0.41)	0.003*
**Dynamic Reconfiguration Measures (Whole-brain average)**^**a**^			
Flexibility	0.15 (0.01)	0.16 (0.01)	0.036*
Promiscuity	0.56 (0.02)	0.55 (0.03)	0.461
Disjointedness	2.36e-03 (1.60e-04)	2.25e-03 (2.07e-04)	0.036*
Cohesion	0.15 (0.01)	0.15 (0.01)	0.036*

^a^Reconfiguration measures are derived from the sliding window length of 19 TRs, with the brain parcellated and segmented based on Schaefer’s cortical and Melbourne’s subcortical atlas.

^†^Wilcoxon signed-rank test.

The Benjamini-Hochberg false discovery rate method was used to correct for multiple comparisons.

To investigate how one-year changes in DaT availability relate to changes in reconfiguration measures, we employed a Bayesian bivariate latent change score model. This model estimates the latent correlation (*ρ*) between two latent variables: baseline-to-year one changes in reconfiguration measures (whole-brain average and network level) and the changes in DaTscan striatal binding ratios. Because medication was initiated between baseline and follow-up in a subset of follow-up participants, medication status was included as a predictor of both latent change scores to account for its potential influence on longitudinal changes in DaT availability and reconfiguration measures. An association between latent variables was considered significant if zero was not included in the 95% posterior credible intervals (CI). After taking into account the diagnostic model fit measures, we observed a significant negative latent correlation for the changes in striatum average DaT availability and changes in average flexibility and cohesion (For SW19: flexibility: *ρ* = −0.347 [95% *CI* = −0.681, −0.012], Posterior predictive *p*-value (*PPP*) = 0.605, Bayesian Root Mean Square Error of Approximation (*BRMSEA*) = 0.059, $\hat{R}\,$ = 1.000; cohesion:*ρ* = −0.339 [95% *CI* = −0.676, −0.003], *PPP* = 0.600, *BRMSEA* = 0.062, $\hat{R}\,$ = 1.000). Of the subregional measures, only changes in caudate average DaT availability were negatively correlated with changes in average flexibility and cohesion (For SW19: flexibility: *ρ* = −0.427 [95% *CI* = −0.745, −0.109], *PPP* = 0.613, *BRMSEA* = 0.057, $\hat{R}$ = 1.000; cohesion:*ρ* = −0.423 [95% *CI* = −0.745, −0.101], *PPP* = 0.619, *BRMSEA* = 0.058, $\hat{R}\,$ = 1.000). In other words, in this sample, greater dopaminergic decline, particularly in caudate, was associated with an increase in whole-brain average flexibility (Fig. 4b) and cohesion. Similar negative associations were observed at the network level, where one-year changes in both the striatum and caudate average DaT availability were negatively correlated with changes in flexibility and cohesion in the DAN, SAL, and VIS networks. The effects were generally robust to the choice of sliding window length (Suppl. Table 9).

Given that more than half of the follow-up sample had started L-DOPA before their one-year follow-up (19 medicated vs. 10 unmedicated), we conducted a post-hoc analysis to examine whether being medicated influenced the associations between DaT availability and reconfiguration measures. We fitted a linear mixed-effects model, including the interaction term between DaT availability and medication status, adjusting for time (baseline vs. year 1), age, sex, and baseline MDS-UPDRS part 3 scores, and accounting for repeated measures with a random intercept per subject. We found a significant interaction between DaT availability and medication status: all DMN reconfiguration measures and disjointedness in the SUB network showed medication-dependent associations with both the striatum and caudate average measures of DaT availability across all sliding window lengths (Fig. 4c, Suppl. Table 10). In other words, initiating dopamine replacement therapy seems to modify the association between DaT availability and network reconfiguration measures by reversing the direction of association.

## Discussion

This study aimed to investigate how striatal DaT availability, as a measure of nigrostriatal dopaminergic integrity, relates to dynamic functional brain network reconfiguration in drug-naive Parkinson’s disease. Patients exhibited reduced network flexibility, promiscuity, and cohesion in the default-mode network, indicating spatially specific alterations. DaT availability was negatively associated with visual network promiscuity but positively associated with limbic network flexibility and cohesion, highlighting network-dependent effects of dopamine loss. While motor impairment was linked to DaT availability, cognitive function was associated with dynamic network reconfiguration in the dorsal attention network, supporting a dissociation between dopamine’s effects on motor and cognitive performance. Follow-up data revealed increases in network flexibility, which were negatively associated with the changes in DaT availability and modulated by dopaminergic medication, suggesting that network dynamics adapt to disease progression and treatment effects over time.

Our findings extend previous research in medicated Parkinson’s disease that linked changes in dopamine to changes in brain network dynamics^26^. We show that, even early in the disease and before treatment, DMN nodes already exhibit reduced module switching compared to healthy controls, a pattern that may compromise cognitive abilities. Importantly, all effects were robust across the three window lengths (SW15, SW19 and SW23) and the two cortical parcellations (Schaefer and Glasser) tested, indicating that the observed alterations in DMN reconfiguration do not depend on a specific parameter choice. The DMN plays a key role in cross-network interactions, with the salience network acting as a switch^32,33^. Meta-analyses consistently report DMN disruptions in Parkinson’s disease^7,34^, and our results add a novel, dynamic perspective; DMN regions switch communities less often, suggesting early network rigidity.

Previous studies investigating dynamic functional connectivity in Parkinson’s disease primarily used K-means clustering, which relies on a predefined number of connectivity “states” identified by pooling data across all individuals^17^. Although this approach quantifies how often patients transition between states and how long they dwell in each state, it does not capture individualized node- and network-level dynamics. Findings are mixed, with some reporting an increase^35,36^, while others report no significant differences or even reduced state transitions in Parkinson’s disease^37,38^. In contrast, measures such as flexibility capture continuous, subject-level reconfiguration of brain regions over time, allowing a more sensitive assessment of how dopamine loss influences dynamic neural organisation.

At the biological level, altered patterns of dynamic network reconfiguration are thought to reflect changes in the brain’s capacity for adaptive communication. In healthy systems, temporally flexible network reorganization supports learning, attention, and cognitive control, enabling brain regions to dynamically integrate and segregate according to task demands. Within this framework, measures such as flexibility, which quantifies how frequently a node changes its community allegiance, have been interpreted as markers of adaptive integration and cognitive flexibility^20^. However, the interpretation of dynamic reconfiguration metrics is not unidirectional. While reduced flexibility may reflect reduced network adaptability^20,23^, elevated flexibility may instead signal network instability or inefficient communication^25,26^. Neuromodulatory systems in the brain play a central role in reshaping functional interactions within and between large-scale networks, optimizing cognitive function^39^. Consequently, dopamine depletion, as in Parkinson’s disease, may lead to either less adaptability or compensatory instability, resulting in less efficient large-scale communication among brain regions. Within this perspective, alterations in dynamic reconfiguration metrics should be interpreted as reflecting changes in network adaptability rather than inherently adaptive or maladaptive processes. Notably, the strong correspondence of whole-brain average flexibility and cohesion across parcellations suggests that these measures capture relatively robust, atlas-independent properties of large-scale network reconfiguration dynamics, whereas other metrics (i.e., promiscuity and disjointedness) appear more sensitive to parcellation choice.

DaT availability showed opposing associations across networks. It was negatively associated with visual promiscuity but positively with limbic network flexibility and cohesion, highlighting network-dependent effects of dopamine loss. These findings align with the idea that different brain networks have distinct optimal dopamine levels^10^. Specifically, the visual network may require a different optimal dopamine range for visual processing than the limbic network, which governs emotion regulation, social interaction, and memory function^40^. This may account for dopaminergic therapy’s variable effects across behavioral domains^8,15,41^, including the dissociation between task-switching and reward-based learning^33^. In line with this view, a recent study by Asendorf et al.^35^ using K-means clustering showed that the number of transitions between dynamic connectivity states correlated with DaT availability in the putamen and caudate. Our multilayer community detection approach extends these findings by capturing subject-specific, nodal, and network-level reconfigurations that remain inaccessible in state-based frameworks.

While DaT availability is a strong predictor of motor deficits in Parkinson’s disease^42,43^, a link confirmed in our analysis, its relationship with cognition remains less clear. DaT availability was linked to specific cognitive domains such as memory and attention^44,45^, but evidence connecting it to general cognitive function remains limited^46^. Our findings suggest that resting-state dynamic network reconfiguration measures may serve as promising biomarkers of cognitive function in Parkinson’s disease, even in recently diagnosed drug-naive patients. General cognition (i.e., MoCA) was related to overall network flexibility, and particularly to flexibility in the dorsal attention network. Attention networks play a central role in coordinating goal-directed and stimulus-driven processes^47^ and an fMRI meta-analytic coactivation model has shown that mild cognitive impairment in Parkinson’s disease seems to converge on the attention network^48^. These associations highlight that reduced dynamic reconfiguration within the nodes of this network may constitute a sensitive marker for emerging cognitive impairment. Future studies could relate dynamic functional connectivity measures to distinct cognitive symptoms like attention/executive function and visuospatial abilities, which are among the earliest cognitive impairments in Parkinson’s disease^49,50^.

Our post-hoc Trail Making Test analyses support this domain-specific interpretation. Flexibility in dorsal attention and control networks was associated with TMTB-A, but not with TMT-A, suggesting that these dynamic measures relate more strongly to executive control and set-shifting than to processing speed or basic visuospatial scanning. This pattern is conceptually consistent with the known functional role of dorsal attention and control networks in top-down attention, cognitive control, and flexible task-set updating^47^.

Although we did not observe significant associations between motor impairment and dynamic network reconfiguration at the whole-brain or network-average level, some nodal associations were identified. However, these were significant only in the Glasser parcellation and were not reproduced using the Schaefer atlas, indicating that they are not robust to parcellation choice.

Differences between parcellation schemes are also important to consider when interpreting nodal findings. Although exact parcel-to-parcel correspondence between the Schaefer and Glasser atlases is not feasible because they differ in parcel definition, size, and boundaries, the broader pattern of results was nevertheless consistent at the systems level. In particular, flexibility and cohesion showed overlapping anatomical associations across parcellations and across levels of analysis, in particular in group differences, associations with DaT availability and cognitive correlates, suggesting that the main signal is not driven by the atlas choice. This interpretation is further supported by the observation of a high cross-parcellation correlation in whole-brain average flexibility and cohesion, whereas promiscuity was less strongly aligned, indicating that flexibility and cohesion may capture reconfiguration aspects that are more robust to parcellation choice. Moreover, Glasser’s multimodal parcellation aims to delineate finer cortical areas, whereas Schaefer’s atlas is explicitly organized around large-scale functional network structure. Thus, atlas-dependent differences may arise because finer areal subdivision can increase sensitivity to more spatially localized effects, while network-oriented parcellations may better capture broader systems-level patterns^27,29^. At the same time, these differences highlight a general limitation of atlas-based approaches as fixed group atlases may not fully capture the interindividual variability in cortical organization^51^. Future studies using individualized or subject-specific parcellations may improve anatomical precision, reduce boundary mismatch, and provide a more reliable characterization of how dynamic network reconfiguration changes in Parkinson’s disease.

Although Parkinson’s disease primarily affects subcortical dopaminergic systems, we observed relatively few significant subcortical findings. Our dynamic reconfiguration measures capture how regions change their community affiliation within the whole-brain network over time, rather than regional dysfunction in isolation. In this framework, the dopamine-related pathology may be expressed more strongly through its effects on large-scale cortical reorganization. In addition, subcortical regions typically have lower signal-to-noise ratio and are vulnerable to partial-volume and registration mismatch effects. In addition, at the network-level, all subcortical regions were averaged into a single subcortical network, following prior literature^21,24,52^, which improves stability but may also obscure more regional effects.

Dynamic functional brain network reconfiguration may offer a mechanistic account of how large-scale networks adapt to progressive dopamine loss and how medication modulates this process. An increase in flexibility after one year of disease progression was evident at the whole-brain level and in the default-mode, control, limbic, and subcortical networks. This pattern mirrors findings from Shine et al.^26^ showing that patients in later disease stages exhibit higher flexibility in several brain regions during OFF (vs. ON L-DOPA) states. Additionally, by modeling individual change trajectories with a Bayesian latent change score model, we observed that progressive dopamine loss was associated with an increase in flexibility at whole-brain and network levels over one year. However, this longitudinal pattern was not uniform across networks. Although flexibility increased over time at the whole-brain level and in the default-mode, control, limbic, and subcortical networks, its coupling with dopamine loss showed a different pattern at the whole-brain level, and in the visual, dorsal, and salient/ventral attention networks. This dissociation is particularly evident for the limbic network. While lower DaT availability was associated with reduced limbic flexibility at baseline, limbic flexibility increased after one year despite ongoing dopamine decline. Importantly, the latent change model did not reveal a significant association between dopamine loss and changes in limbic network flexibility, suggesting that the longitudinal increase is unlikely to be explained by striatal dopaminergic decline alone. Instead, the observed increase may reflect the influence of medication included in the model, early compensatory mechanisms such as network hyperactivation^53,54^, or changes in other neurotransmitter systems such as the cholinergic system^55,56^.

Nonetheless, the association of dopamine decline with longitudinal changes in dorsal attention, salient/ventral attention, and visual networks remains an important finding. Prior fMRI studies indicate that dopaminergic depletion and therapy reshape large-scale connectivity in a mixture of potentially compensatory and pathological ways, with increased coupling in motor circuits sometimes improving performance^57^, and excessive connectivity relating to symptoms such as dyskinesia^58^. In this context, the observed increase in dynamic reconfiguration with dopamine loss in our longitudinal subsample should be interpreted cautiously as an indicator of altered network adaptation, rather than as inherently beneficial or detrimental.

Interestingly, the longitudinal changes in DaT availability and changes in reconfiguration measures were not uniform across striatal regions: decreases in caudate average, but not putamen average DaT availability, were associated with increased flexibility in dorsal and ventral attention networks. Cross-sectionally, we had already observed that lower flexibility in the dorsal attention network was related to poorer cognition in drug-naive patients. In the longitudinal sample, MoCA scores remained stable over one year, indicating that the increase in flexibility occurred in the absence of measurable cognitive decline. Given that caudate dopamine loss, rather than the putamen, has been consistently linked to cognitive rather than motor deficits^59,60^, the observed coupling between progressive caudate dopamine loss and increasing attention network flexibility may indicate an adaptive reconfiguration in response to dopaminergic depletion. Alternatively, it might represent early network instability preceding cognitive decline. Together, these findings suggest that dynamic network reconfiguration, particularly flexibility within attention networks, may serve as sensitive markers of dopaminergic modulation of cognition in Parkinson’s disease.

Dopamine replacement therapy significantly influenced the relationship between DaT availability and network dynamics. In medicated patients, the association reversed relative to the unmedicated state and more closely resembled that of healthy controls, particularly within the DMN. These findings suggest that dopaminergic medication modulates and may partially restore large-scale network dynamics disrupted in early disease. In line with this, in a recent task-based fMRI study, Lee et al.^61^ demonstrated that dopaminergic medication dynamically reshapes brain state engagement during working memory processing in a dose-dependent, inverted U-shaped manner. Similar dose-dependent or context-specific effects have been reported in other studies showing restoration of DMN functional properties after treatment^62,63^, highlighting the potential of dynamic functional connectivity measures as promising biomarkers of treatment response, supporting their further exploration in future studies. Although based on a longitudinal case-control design rather than a randomised controlled trial, our study offers a first step toward understanding how dopaminergic therapy influences network reconfiguration over time, warranting validation in larger and more controlled longitudinal designs.

Several limitations should be considered. The longitudinal analyses were based on a smaller subset of participants with repeat imaging at one year, and many of these patients initiated dopaminergic treatment during this interval. Although appropriate statistical models with satisfactory model fit were used, larger longitudinal cohorts with more controlled treatment conditions will be important to further disentangle medication-related and disease progression-related effects on network reconfiguration. Cognitive associations were primarily assessed using the MoCA, a global screening measure, and the TMT, which captures processing speed, visual attention, and executive function. Future studies that combine dynamic network metrics with more extensive domain-specific neuropsychological testing may help to further clarify the specific cognitive processes linked to network reconfiguration. In addition, the study relied on multi-site imaging data from the PPMI cohort; although standardized acquisition protocols and containerized preprocessing pipelines were used to ensure consistent processing across sites, future work may further benefit from explicit harmonization approaches and independent replication cohorts to confirm the generalizability of these findings. Finally, although DaTscan-derived SBR reflects dopamine transporter availability and presynaptic nigrostriatal dopaminergic integrity, it does not directly measure dopamine levels or release. Future studies using more direct measures of dopaminergic function may further clarify its relationship with dynamic network reconfiguration in healthy and disease brains.

In summary, our study demonstrated that patients with early-stage Parkinson’s disease, prior to medication, already show altered network dynamics, highlighting network reconfiguration as a core feature of the disease. Using multilayer community detection, we provide novel insights into how nigrostriatal dopaminergic integrity impacts the brain’s dynamic functional reconfiguration. We show that striatal dopamine loss affects dynamics in a network-dependent manner, with opposite effects across visual and limbic systems, consistent with the notion that distinct networks operate within different optimal dopaminergic ranges. As dopamine loss progresses, flexibility increases over time, particularly in the default-mode, control, limbic, and subcortical networks, suggesting a dynamic adaptation. Progressive caudate dopamine decline, but not putaminal, was specifically linked to changes in attention network flexibility, pointing to a sensitive neural marker of cognitive vulnerability that may precede a measurable decline in cognition. Dopaminergic medication further modulated these dynamics, partially reversing associations to resemble those of healthy controls. Together, these findings underscore the value of functional network dynamics as markers of dopamine-related brain changes and underscore their potential as promising biomarkers for cognitive function and treatment response in Parkinson’s disease.

## Methods

### Participants

We included data from 143 patients and 20 healthy controls from the Parkinson’s Progression Markers Initiative (PPMI) dataset (https://ppmi-info.org)^64^. At baseline, patients had a Parkinson’s disease diagnosis for ≤ 2 years and were drug-naive. For full inclusion/exclusion criteria, see https://www.ppmi-info.org/study-design/research-documents-and-sops.

We selected subjects with both DaTscan and rsfMRI at baseline, the latter acquired in two opposing phase-encoding directions, ensuring sufficient subjects with high-quality, homogeneous rsfMRI for the analysis^17^. Thirty patients had DaTscan and rsfMRI data available at a one-year follow-up; after exclusion of one follow-up sample due to excessive motion, 29 patients were included in the longitudinal analysis. PPMI is a multi-site observational study conducted in accordance with the Declaration of Helsinki and Good Clinical Practice guidelines. The study protocol and informed consent documents were approved by the Institutional Review Board (IRB)/Ethics Committee at each participating site, and written informed consent was obtained from all participants (ClinicalTrials.gov identifier: NCT01141023)^64^. Seven patients were excluded due to excessive motion, resulting in the final sample of 156 participants (136 drug-naive early-stage Parkinson’s disease patients and 20 healthy controls; Table 1).

### Clinical assessments

We used baseline and one-year follow-up measurements of the Movement Disorder Society – Unified Parkinson’s Disease Rating Scale (MDS-UPDRS) part 3 score^65^ and Montreal Cognitive Assessment (MoCA) scores as motor and cognitive function measures, respectively. To further examine attention and executive function, we used the Trail Making Test (TMT). Time of completion of TMT-A was included as a measure of processing speed and visual attention, and the time difference of TMT-B completion and TMT-A (TMTB-A) as a measure of executive function.

### Dopamine transporter scans

DaTscan (123I-FP-CIT SPECT) imaging was performed at PPMI imaging centers. Striatal binding ratios of left/right caudate and putamen were provided by PPMI. In this dataset, striatal binding ratio is defined as (target region/reference region) − 1, using the occipital cortex as the reference region. We used DaTscan measures at baseline and one-year follow-up as an index for nigrostriatal dopaminergic integrity. For analysis, mean binding values were computed separately for the caudate, putamen, and their combined striatal average at each visit.

### MRI acquisition and preprocessing

For each participant, T1-weighted anatomical and rsfMRI scans were acquired as part of the same PPMI MRI protocol on 3 T scanners. T1-weighted scans were acquired with 1 mm isotropic voxel-size, number of slices = 192, and FOV = 256 mm (TR/TE values varied by site and scanner). Resting-state functional MRI T2*-weighted echo planar imaging data were acquired with the same acquisition parameters across participants: TR/TE = 2500/30 ms, phase-encoding directions = RL/LR, with isotropic voxel-size = 3.5 mm, flip angle = 80°, acquisition matrix = 64 × 64, FoV = 224 mm, 240 volumes (acquisition time: approx. 10 min). Subjects were instructed to keep their eyes open and remain still.

Raw DICOM data were converted to the BIDS format using HeudiConv v0.13.1^66^ [RRID: SCR_017427]. To ensure reproducibility and consistency of preprocessing, we utilized the containerized preprocessing tools fMRIPrep v20.2.7^67^ [RRID: SCR_016216] [based on Nipype 1.7.0^68^; RRID: SCR_002502)] and XCP-D v0.5.0^69^ [RRID: SCR_017427]. These pipelines are widely used in the neuroimaging community and integrate multiple established tools (e.g., ANTs, FreeSurfer, FSL, AFNI) within a single automated workflow, thereby avoiding manual combination of separate software packages. A detailed description of preprocessing steps, the generated boilerplates from fMRIPrep and XCP-D, is provided in the supplementary materials. In brief, anatomical preprocessing included N4 bias correction, skull stripping, and tissue segmentation using FreeSurfer (version 6.0.1)^70^, and nonlinear registration to the MNI152NLin6Asym space^71^. Functional preprocessing comprised distortion, motion, and slice-timing correction, followed by co-registration to the T1-weighted image and then normalization to MNI152NLin6Asym space. No spatial smoothing was applied prior to denoising. Post-processing with XCP-D implemented the *acompcor_gsr* strategy, which included regression of ten aCompCor^72^ components from white matter and cerebrospinal fluid masks (five components each), six motion parameters and their temporal derivatives, and the global signal. We chose this approach because including global signal regression alongside aCompCor improved the denoising strategy^73^. Moreover, aCompCor pipeline was one of the two denoising strategies resulting in no association between node flexibility and subject motion among several denoising strategies in an earlier dynamic functional connectivity analysis^74^. Denoising was followed by temporal filtering (0.008–0.1 Hz) and recensoring of high-motion volumes (frame-wise displacement > 0.5 mm). The final denoised BOLD data were not spatially smoothed and were used to extract regional time series from the Schaefer 400-parcel cortical atlas (17-network version)^27^ and 50 subcortical regions of the Melbourne subcortical atlas^28^, ensuring comprehensive cortical and subcortical coverage. To evaluate the robustness of the nodal findings to the chosen cortical parcellation, an additional analyses at the nodal level were conducted using Glasser’s multi-modal parcellation atlas^29^.

### Dynamic network reconfiguration measures

Subjects with excessive motion (frame-wise displacement > 0.5 mm for > 30% of time points) were excluded (7 at baseline, one at follow-up). Additionally, regions with voxel coverage < 50% in at least one subject were removed to ensure uniform matrix sizes across participants, resulting in 423 areas at baseline and 438 at follow-up.

**Fig. 5:**
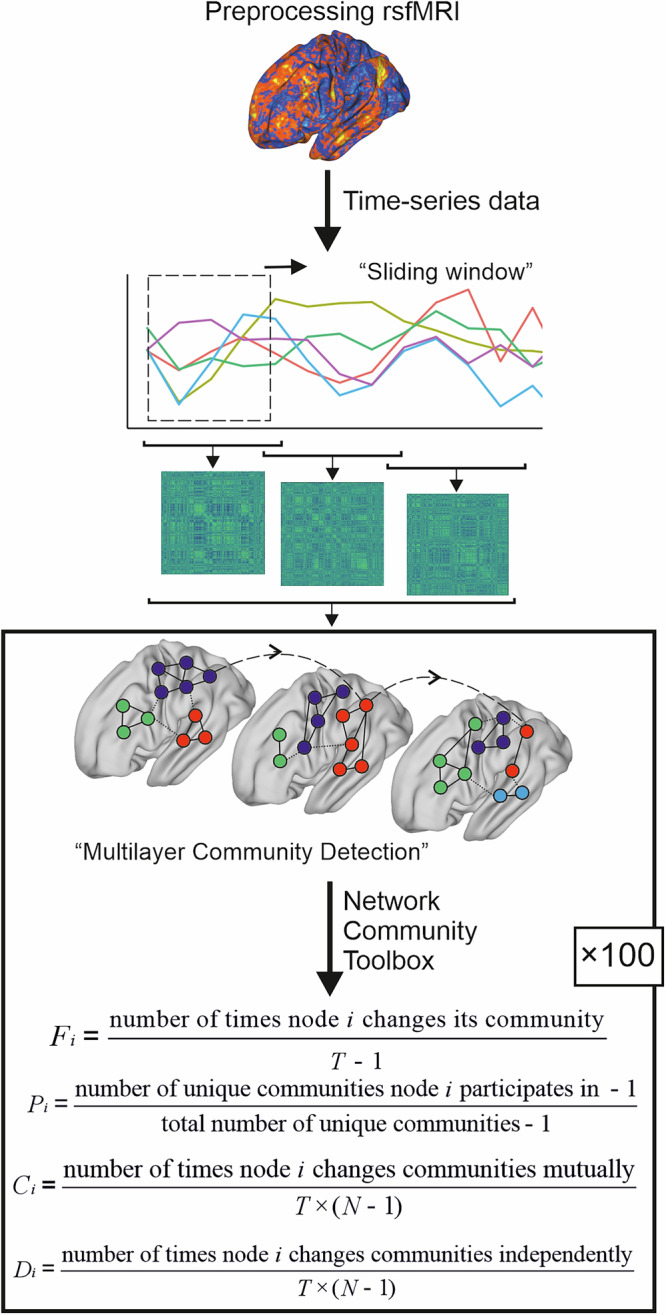
Overview of the imaging analysis pipeline. After preprocessing the resting-state functional MRI data, a sliding window approach was used on whole-brain time series data, generating a multilayer temporal structure. The pairwise functional connectivity was calculated within each window, and a multilayer community detection algorithm (GenLouvain) was applied to identify temporally changing modules shown by different colors. Dynamic network reconfiguration measures were then calculated using the Network Community Toolbox. Due to slight changes in community assignment of each node in each iteration of the GenLouvain algorithm, the last two steps were iteratively performed 100 times, and the dynamic network reconfiguration measures were averaged. The formulas to calculate Flexibility ${(F}_{i})$, Promiscuity (${P}_{i}$), Cohesion (*C*_*i*_), and Disjointedness (*D*_*i*_) are provided in the figure. “N” is the total number of nodes, and “T” is the number of windows.

To capture transient changes in connectivity, we applied a Gaussian sliding window approach using Comet toolbox^75^. We used three window lengths: 15 (37.5 s), 19 (47.5 s), and 23 (57.5 s) TRs with a step size of one and a standard deviation of 3 TRs. Previous studies suggest an optimal window length of 30–60 seconds as a trade-off between sensitivity to transient changes in connectivity and avoiding false positives^17^. We report the main findings for the 19-TR window, while all analyses were repeated with 15 and 23 TRs, confirming that results were robust across window lengths. Intralayer functional connectivity matrices were then calculated using Pearson’s correlation and were Fisher’s r-to-z transformed. Negative and self-connections were removed prior to community detection, and no additional density thresholding was applied. This approach is consistent with commonly used multilayer dynamic functional connectivity frameworks, where community detection is performed on weighted connectivity matrices without binarization or sparsity thresholding^20,23,24,26,76^. After removing the negative and self-connections, the resulting weighted connectivity matrices were then used as input to the GenLouvain algorithm (https://github.com/GenLouvain/GenLouvain) for multilayer community detection^18,19^. To maintain comparability with prior multilayer dynamic functional connectivity studies, we used the default spatial and temporal resolution parameters of one. This algorithm identifies time-resolved communities and assigns each node to a community in each window (Fig. 5).

The Network Community Toolbox^77^ (http://commdetect.weebly.com/) was then used to calculate the following network reconfiguration measures for each node: Flexibility, Promiscuity, Cohesion, and Disjointedness (Fig. 5). Flexibility measures how often a node changes its community relative to the total number of possible switches^20^. Promiscuity assesses the fraction of all possible communities joined by a node^78^. Cohesion quantifies nodes changing communities together. High cohesion implies coordinated shifts among nodes. Disjointedness measures solo community switches^79^. Nodes with high disjointedness tend to change communities independently. The module-detection and reconfiguration-measure calculation processes were repeated 100 times and averaged to account for slight changes in module-to-node assignments across runs of the GenLouvain algorithm.

### Statistical analysis

Statistical analyses were conducted in RStudio^80^ using R version 4.3.1^81^ to assess group differences in dynamic network reconfiguration measures and their relationship with DaT availability and clinical measures. These measures were tested on three levels: global, networks (average over the nodes of the respective networks), and nodal. Included networks were defined based on Schaefer’s seven networks: default-mode (DMN), visual (VIS), somatomotor (SM), dorsal attention (DAN), salient-ventral attention (SAL), limbic (LIMB), and control (CONT)^27^. The subcortical network (SUB) was defined as the average of all subcortical regions. The associations of dynamic network reconfiguration measures with clinical data and DaT availability were assessed using the least trimmed squares robust regression model (*ltsReg* in the “robustbase” library; *α* = 0.85)^82,83^. The LTS estimator bases parameter estimation on the α proportion of observations (in this study 85%), thereby reducing the influence of observations with large residuals in a data-driven manner while retaining all data points in the analysis, making these robust models more resilient to the outliers. In addition, they do not necessarily require the normality assumption of the ordinary linear regression models, and reduce the false-positive likelihood^84,85^. Age and sex were entered in statistical models as covariates. Education was also included as a covariate for statistical tests evaluating cognitive measures. We used Benjamini-Hochberg’s false discovery rate (FDR) correction for multiple comparisons^86^ within each analysis family (i.e., separately by analysis level and dynamic metric). For nodal analyses, correction was performed across all regions ( ~ 450 nodes) within each sliding-window configuration.

To investigate longitudinal changes in DaT availability and reconfiguration measures, we implemented a Bayesian bivariate latent change score model (LCS) using the *blavaan* package^87,88^. Latent change score models^89^ are structural equation models that provide a powerful tool for analyzing longitudinal data. Unlike simple difference scores, LCS models estimate an underlying ‘change factor’ that explains observed changes between time points. By treating change as a latent variable, these models reduce measurement noise and better capture interindividual differences in how DaT availability and reconfiguration measures evolve over time^90^. A Bayesian framework is particularly well-suited for small sample sizes to have more stable correlation estimates in an LCS model and to reduce estimation uncertainty^91,92^. Medication status at follow-up was incorporated into the latent change score model as an exogenous predictor of the latent change factors. Specifically, medication status was modeled as a predictor of change in DaT availability and change in dynamic functional connectivity measures, allowing us to account for potential medication-related influences on longitudinal trajectories. Goodness of fit measures for *blavaan*, namely posterior predictive p-value (PPP), Bayesian variant of the root mean square error of approximation (BRMSEA), and $R$^ as a measure for Markov-Chain convergence were reported for the models. In general, PPP between 0.05 and 0.95 (0.50 as the best), BRMSEA less than 0.08, and $R$^close to 1 (in general, less than 1.01) are indicative of a good fit^88,93,94^.

## Supplementary information

supplementary_materials

## Data Availability

Data used in this project were downloaded from the Parkinson’s Progression Markers Initiative (PPMI) database (https://ppmi-info.org/).
