# Supplementary material for: Dopamine-related alterations in functional brain network dynamic reconfiguration in Parkinson’s disease
Source: NPJ Parkinsons Dis. 2026 Jul 21;12:175. doi: 10.1038/s41531-026-01466-w (PMC13388714; doi:10.1038/s41531-026-01466-w)
Supplement: Supplementary file 1 — supplementary_materials [file 41531_2026_1466_MOESM1_ESM.pdf]

Dopamine-Related Alterations in Functional Brain Network Dynamic Reconfiguration in Parkinson's Disease

AmirHussein Abdolalizadeh, Micha Burkhardt, Paria Jahansa, Carsten Gießing, Karsten Witt, Christiane M. Thiel

Supplementary Materials

MRI analysis tools boilerplates. Boilerplate text generated by fMRIprep and XCP-D.

Supplementary Table 1. Whole-brain average dynamic network reconfiguration measures do not differ between healthy controls and Parkinson's disease patients.

Supplementary Table 2. Mean (SD) of dynamic network reconfiguration measures of networks in healthy controls and Parkinson's disease patients for different sliding window lengths.

Supplementary Table 3. Associations between striatal dopamine transporter availability and dynamic network reconfiguration measures in Parkinson's disease patients.

Supplementary Table 4. Significant associations between striatal dopamine transporter availability and nodal dynamic network reconfiguration measures in Parkinson's disease patients using Schaefer's parcellation.

Supplementary Table 5. Significant associations between striatal dopamine transporter availability and nodal dynamic network reconfiguration measures in Parkinson's disease patients using Glasser's parcellation.

Supplementary Table 6. Associations between MoCA scores and dynamic network reconfiguration measures in Parkinson's disease patients.

Supplementary Table 7. Baseline and one-year dynamic network reconfiguration measures based on the sliding window length of 15 TRs for 29 patients with follow-up data.

Supplementary Table 8. Baseline and one-year visit dynamic network reconfiguration measures based on a sliding window of 23 TRs for 29 patients with follow-up data.

Supplementary Table 9. Bayesian Latent Change Score model results.

Supplementary Table 10. Linear mixed effect model results for dynamic network reconfiguration measures as the dependent variable and the interaction between striatum average dopamine transporter availability and medication as the predictor variables.

Supplementary Figures 1-2. Significant group differences in nodal dynamic network reconfiguration measures between healthy controls and Parkinson's disease patients using Schaefer's cortical parcellation for sliding window lengths of 15 and 23 TRs, respectively.

Supplementary Figures 3-5. Significant group differences in nodal dynamic network reconfiguration measures between healthy controls and Parkinson's disease patients using Glasser's parcellation for sliding window lengths of 15-19-23 TRs.

Supplementary Figures 6-8. Nodal dynamic network reconfiguration measures that were significantly associated with striatum average DaT availability using Schaefer's parcellation for sliding window lengths of 19, 15, and 23 TRs in healthy controls, respectively.

Supplementary Figure 9. Comparison of uncorrected significant findings ($p < 0.001$) across parcellation atlases.

Supplementary Figures 10-11. Nodal dynamic network reconfiguration measures that were significantly associated with the MoCA total score using Schaefer's parcellation for sliding window lengths of 15 and 23 TRs in Parkinson's disease patients.

Supplementary Figure 12-13. Nodal dynamic network reconfiguration measures that were significantly associated with the total MoCA score using Glasser's parcellation for sliding window lengths of 15 and 23 TRs in Parkinson's disease patients.

Supplementary Figure 14. Nodal dynamic network reconfiguration measures that were significantly associated with the MDS-UPDRS part 3 total score for sliding window length of 15 TRs in Parkinson's disease patients, using Schaefer's parcellation.

Supplementary Figures 15-17. Nodal dynamic network reconfiguration measures that were significantly associated with the MDS-UPDRS part 3 total score using Glasser's parcellation for sliding window lengths of 19, 15, and 23 TRs in Parkinson's disease patients, respectively.

Supplementary References.

MRI analysis tools boilerplates. Boilerplate text generated by fMRIprep and XCP-D.

Anatomical Data Preprocessing with fMRIprep

The T1-weighted (T1w) image was corrected for intensity non-uniformity (INU) with N4BiasFieldCorrection,[1] distributed with ANTs 2.3.3,[2] and used as T1w-reference throughout the workflow. The T1w-reference was then skull-stripped with a Nipype implementation of the antsBrainExtraction.sh workflow (from ANTs), using OASIS30ANTs as the target template. Brain tissue segmentation of cerebrospinal fluid (CSF), white matter (WM), and gray-matter (GM) was performed on the brain-extracted T1w using fast (FSL 5.0.9).[3] Brain surfaces were reconstructed using recon-all (FreeSurfer 6.0.1),[4] and the brain mask estimated previously was refined with a custom variation of the method to reconcile ANTs-derived and FreeSurfer-derived segmentations of the cortical gray-matter of Mindboggle.[5] Volume-based spatial normalization to MNI152NLin6Asym standard space was performed through nonlinear registration with antsRegistration (ANTs 2.3.3), using brain-extracted versions of both T1w reference and the T1w template. The following template was selected for spatial normalization: FSL's MNI ICBM 152 non-linear 6th Generation Asymmetric Average Brain Stereotaxic Registration Model [TemplateFlow ID: MNI152NLin6Asym].[6]

Functional Data Preprocessing with fMRIprep

First, a reference volume and its skull-stripped version were generated using a custom methodology of fMRIPrep. A B0-nonuniformity map (or fieldmap) was estimated based on two echo-planar imaging (EPI) references with opposing phase-encoding directions, with 3dQwarp (AFNI 20160207).[7] Based on the estimated susceptibility distortion, a corrected EPI (echo-planar imaging) reference was calculated for a more accurate co-registration with the anatomical reference. The BOLD reference was then co-registered to the T1w reference using bbregister (FreeSurfer) which implements boundary-based registration.[8] Co-registration was configured with six degrees of freedom. Head-motion parameters with respect to the BOLD reference (transformation matrices, and six corresponding rotation and translation parameters) were estimated before any spatiotemporal filtering using mcflirt (FSL 5.0.9).[9] BOLD runs were slice-time corrected to 1.21s (0.5 of slice acquisition range 0s-2.42s) using 3dTshift from AFNI 20160207.[7] The BOLD time series were resampled onto their original, native space by applying a single, composite transform to correct for head-motion and susceptibility distortions. These resampled BOLD time series will be referred to as preprocessed BOLD in original space, or just preprocessed BOLD. The BOLD time series were resampled into MNI152NLin6Asym standard space, correspondingly generating the spatially-normalized, preprocessed BOLD run. Several confounding time series were calculated based on the preprocessed BOLD: framewise displacement (FD), DVARS and three region-wise global signals. FD was computed using two formulations following Power (absolute sum of relative

motions)[10] and Jenkinson (relative root mean square displacement between affines).[9] FD and DVARS are calculated, both using their implementations in Nipype (following the definitions by Power et al.[10]). The three global signals are extracted within the CSF, the WM, and the whole-brain masks. Additionally, a set of physiological regressors were extracted to allow for component-based noise correction (CompCor)[11] Principal components are estimated after high-pass filtering the preprocessed BOLD time series (using a discrete cosine filter with 128s cut-off) for the two CompCor variants: temporal (tCompCor) and anatomical (aCompCor). tCompCor components are then calculated from the top 2% variable voxels within the brain mask. For aCompCor, three probabilistic masks (CSF, WM and combined CSF+WM) are generated in anatomical space. The implementation differs from that of Behzadi et al.[11] in that instead of eroding the masks by 2 pixels on BOLD space, the aCompCor masks are subtracted from a mask of pixels that likely contain a volume fraction of GM. This mask is obtained by dilating a GM mask extracted from the FreeSurfer's aseg segmentation, and it ensures components are not extracted from voxels containing a minimal fraction of GM. Finally, these masks are resampled into BOLD space and binarized by thresholding at 0.99 (as in the original implementation). Components are also calculated separately within the WM and CSF masks. For each CompCor decomposition, the k components with the largest singular values are retained, such that the retained components' time series are sufficient to explain 50 percent of variance across the nuisance mask (CSF, WM, combined, or temporal). The remaining components are dropped from consideration. The head-motion estimates calculated in the correction step were also placed within the corresponding confounds file. The confound time series derived from head motion estimates and global signals were expanded with the inclusion of temporal derivatives and quadratic terms for each.[12] Frames that exceeded a threshold of 0.5 mm FD or 1.5 standardised DVARS were annotated as motion outliers. All resamplings can be performed with a single interpolation step by composing all the pertinent transformations (i.e. head-motion transform matrices, susceptibility distortion correction, and co-registrations to anatomical and output spaces). Gridded (volumetric) resamplings were performed using antsApplyTransforms (ANTs), configured with Lanczos interpolation to minimize the smoothing effects of other kernels [13]. Non-gridded (surface) resamplings were performed using mri_vol2surf (FreeSurfer).

Many internal operations of fMRIPrep use Nilearn 0.6.2,[14] mostly within the functional processing workflow. For more details of the pipeline, see the section corresponding to workflows in fMRIPrep's documentation (https://fmriprep.org/en/20.2.7/workflows.html).

Postprocessing the fMRIprep Output with XCP-D

The eXtensible Connectivity Pipeline - DCAN (XCP-D) v0.5.0[15] was used to post-process the outputs of fMRIPrep (https://xcp-d.readthedocs.io/en/0.5.0/). XCP-D was built with Nipype version 1.8.6.[16] For each functional data set, the following post-processing was performed. Volumes

with framewise displacement greater than 0.5 mm were flagged as high-motion outliers for the sake of later censoring.[10] Nuisance regressors were selected according to the 'acompcor_gsr' strategy. We chose this approach because the inclusion of global signal regression (GSR) in addition to aCompCor was beneficial in improving the denoising strategies.[17] Moreover, aCompCor pipeline was the only denoising strategy resulting in no association between node flexibility and subject motion among several denoising strategies in dynamic functional connectivity analysis.[18] The top 5 aCompCor principal components from the white matter and cerebrospinal fluid compartments were selected as nuisance regressors,[11] along with the six motion parameters and their temporal derivatives, and mean global signal.[12,19] As the aCompCor regressors were generated on high-pass filtered data, the associated cosine basis regressors were included. This has the effect of high-pass filtering the data as well. Finally, linear trend and intercept terms were added to the regressors prior to denoising. Nuisance regressors were regressed from the BOLD data using linear regression, as implemented in Nilearn. Any volumes censored earlier in the workflow were then interpolated in the residual time series produced by the regression. The interpolated time series were then band-pass filtered using a second-order Butterworth filter, in order to retain signals between 0.008-0.1 Hz. The filtered, interpolated time series were then re-censored to remove high-motion outlier volumes. The denoised BOLD was not smoothed.

Processed functional time series were extracted from the residual BOLD signal with Nilearn's *NiftiLabelsMasker* for the following atlases in the next steps: the Schaefer 17-network 400[20], Glasser's multi-modal parcellation,(Glasser et al. 2016) and the Tian subcortical atlas 50.[21] In cases of partial coverage, uncovered voxels (values of all zeros or NaNs) were either ignored (when the parcel had >50.0% coverage) or were set to zero (when the parcel had <50.0% coverage). Many internal operations of XCP-D use AFNI,[7,22] ANTS,[2] TemplateFlow version 0.8.1,[23] matplotlib version 3.4.3,[24] Nibabel version 5.0.1,[25] Nilearn version 0.10.1,[14] numpy version 1.22.4,[26] pybids version 0.15.5,[27] and scipy version 1.10.1.[28] For more details, see the XCP-D website (https://xcp-d.readthedocs.io).

Supplementary Table 1 Whole-brain average dynamic network reconfiguration measures do not differ between healthy controls and Parkinson's disease patients. No significant group differences in average dynamic network reconfiguration measures were noted for the Gaussian sliding window (GSW) lengths of 15 and 23 TRs. Variables with † had non-normal distribution, thus they are reported as median [Interquartile range] and have been tested with non-parametric tests.

	**Healthy Controls (n = 20)**	**Parkinson's Disease (n = 136)**	**Corrected p-value**
**GSW = 15 TRs**			
Flexibility	0.16 (0.01)	0.16 (0.01)	0.442
Promiscuity	0.56 (0.03)	0.56 (0.02)	0.874
Disjointedness	2.42e-03 (1.83e-04)	2.33e-03 (1.74e-04)	0.190
Cohesion	0.16 (0.01)	0.16 (0.01)	0.442
**GSW = 23 TRs**			
Flexibility†	0.16 [0.15, 0.16]	0.15 [0.15, 0.16]	0.458
Promiscuity	0.56 (0.03)	0.56 (0.03)	0.928
Disjointedness	2.42e-03 (1.93e-04)	2.35e-03 (1.79e-04)	0.458
Cohesion†	0.15 [0.15, 0.16]	0.15 [0.14, 0.16]	0.458

Supplementary Table 2 Mean (SD) of dynamic network reconfiguration measures of networks in healthy controls and Parkinson's disease patients for different sliding window lengths. The p-values are based on a robust regression model with age and sex as covariates. Networks are Vis = Visual, SM = Somatomotor, DAN = Dorsal Attention, SAL = Ventral Attention - Salient, LIMB = Limbic, CONT = Control, DMN = Default mode, SUB = Subcorticals.

	**Healthy Controls n = 20**	**Parkinson's Disease n = 136**	***p*-value**	**FDR-Corrected *p*-value**
**GSW = 19 TRs**				
**Flexibility**				
VIS	0.155 (0.014)	0.152 (0.016)	1.000	1
SM	0.161 (0.012)	0.161 (0.016)	0.729	0.833
DAN	0.153 (0.015)	0.15 (0.014)	0.244	0.59
SAL	0.159 (0.013)	0.156 (0.013)	0.599	0.778
LIMB	0.17 (0.013)	0.167 (0.014)	0.178	0.518
CONT	0.155 (0.014)	0.149 (0.015)	0.103	0.441
DMN	0.151 (0.016)	0.143 (0.016)	< 0.001	**0.002**
SUB	0.174 (0.012)	0.171 (0.011)	0.258	0.59
**Promiscuity**				
VIS	0.555 (0.028)	0.556 (0.035)	0.558	0.778
SM	0.563 (0.03)	0.574 (0.031)	0.040	0.317
DAN	0.544 (0.035)	0.553 (0.03)	0.501	0.778
SAL	0.556 (0.028)	0.563 (0.03)	0.378	0.672
LIMB	0.579 (0.033)	0.58 (0.028)	0.529	0.778
CONT	0.551 (0.033)	0.549 (0.032)	0.782	0.863
DMN	0.547 (0.037)	0.534 (0.035)	0.004	**0.039**
SUB	0.587 (0.026)	0.588 (0.026)	0.693	0.821
**Disjointedness**				
VIS	0.002 (3.68e-04)	0.002 (3.87e-04)	0.926	0.988
SM	0.002 (3.17e-04)	0.002 (3.42e-04)	0.298	0.597
DAN	0.002 (3.1e-04)	0.002 (3.64e-04)	0.155	0.518
SAL	0.002 (3.47e-04)	0.002 (3.74e-04)	0.066	0.423

LIMB	0.003 (0.001)	0.003 (0.001)	0.434	0.73
CONT	0.002 (3.98e-04)	0.002 (3.72e-04)	0.168	0.518
DMN	0.002 (3.65e-04)	0.002 (3.18e-04)	0.092	0.441
SUB	0.003 (6.96e-04)	0.003 (4.61e-04)	0.625	0.778
**Cohesion**				
VIS	0.153 (0.014)	0.15 (0.016)	0.967	0.999
SM	0.158 (0.012)	0.159 (0.016)	0.632	0.778
DAN	0.15 (0.015)	0.148 (0.014)	0.253	0.59
SAL	0.157 (0.013)	0.154 (0.013)	0.625	0.778
LIMB	0.167 (0.012)	0.164 (0.014)	0.326	0.614
CONT	0.152 (0.014)	0.147 (0.015)	0.110	0.441
DMN	0.149 (0.016)	0.141 (0.016)	< 0.001	**0.002**
SUB	0.17 (0.012)	0.167 (0.011)	0.285	0.597
**GSW = 15 TRs**				
**Flexibility**				
VIS	0.158 (0.014)	0.155 (0.016)	0.737	0.773
SM	0.164 (0.012)	0.165 (0.015)	0.585	0.693
DAN	0.156 (0.015)	0.154 (0.014)	0.209	0.394
SAL	0.162 (0.013)	0.16 (0.013)	0.325	0.485
LIMB	0.173 (0.013)	0.17 (0.014)	0.204	0.394
CONT	0.158 (0.013)	0.153 (0.015)	0.068	0.279
DMN	0.154 (0.017)	0.146 (0.016)	< 0.001	**0.001**
SUB	0.176 (0.012)	0.174 (0.011)	0.120	0.342
**Promiscuity**				
VIS	0.555 (0.029)	0.555 (0.036)	0.798	0.798
SM	0.564 (0.028)	0.573 (0.03)	0.121	0.342
DAN	0.547 (0.037)	0.551 (0.029)	0.629	0.719
SAL	0.557 (0.031)	0.561 (0.03)	0.432	0.553
LIMB	0.581 (0.034)	0.577 (0.028)	0.040	0.211
CONT	0.555 (0.032)	0.547 (0.03)	0.138	0.342
DMN	0.55 (0.038)	0.533 (0.034)	< 0.001	**0.004**

SUB	0.587 (0.026)	0.585 (0.025)	0.380	0.507
**Disjointedness**				
VIS	0.002 (4.08e-04)	0.002 (3.86e-04)	0.562	0.691
SM	0.002 (3.38e-04)	0.002 (3.43e-04)	0.334	0.485
DAN	0.002 (3.21e-04)	0.002 (3.54e-04)	0.288	0.485
SAL	0.002 (3.35e-04)	0.002 (3.67e-04)	0.013	0.083
LIMB	0.003 (0.001)	0.003 (0.001)	0.312	0.485
CONT	0.002 (3.59e-04)	0.002 (3.54e-04)	0.078	0.279
DMN	0.002 (3.38e-04)	0.002 (2.94e-04)	0.011	0.083
SUB	0.003 (6.67e-04)	0.003 (4.55e-04)	0.274	0.485
**Cohesion**				
VIS	0.156 (0.014)	0.153 (0.016)	0.719	0.773
SM	0.162 (0.012)	0.163 (0.015)	0.749	0.773
DAN	0.154 (0.014)	0.152 (0.013)	0.189	0.394
SAL	0.16 (0.013)	0.157 (0.013)	0.352	0.49
LIMB	0.17 (0.013)	0.167 (0.014)	0.186	0.394
CONT	0.156 (0.013)	0.15 (0.015)	0.072	0.279
DMN	0.152 (0.016)	0.144 (0.015)	< 0.001	**0.001**
SUB	0.173 (0.012)	0.171 (0.011)	0.139	0.342
**GSW = 23 TRs**				
**Flexibility**				
VIS	0.154 (0.014)	0.152 (0.016)	0.990	0.99
SM	0.16 (0.013)	0.161 (0.016)	0.571	0.802
DAN	0.152 (0.015)	0.15 (0.014)	0.251	0.562
SAL	0.159 (0.013)	0.156 (0.013)	0.642	0.848
LIMB	0.169 (0.012)	0.167 (0.014)	0.430	0.655
CONT	0.154 (0.013)	0.149 (0.015)	0.125	0.434
DMN	0.151 (0.016)	0.143 (0.016)	< 0.001	**0.004**
SUB	0.173 (0.012)	0.17 (0.012)	0.374	0.645
**Promiscuity**				
VIS	0.558 (0.03)	0.557 (0.036)	0.860	0.931

SM	0.567 (0.028)	0.575 (0.033)	0.085	0.434
DAN	0.547 (0.036)	0.553 (0.032)	0.863	0.931
SAL	0.561 (0.027)	0.564 (0.031)	0.710	0.848
LIMB	0.583 (0.031)	0.581 (0.03)	0.240	0.562
CONT	0.554 (0.034)	0.55 (0.033)	0.243	0.562
DMN	0.551 (0.041)	0.534 (0.037)	< 0.001	**0.004**
SUB	0.59 (0.026)	0.589 (0.027)	0.400	0.645
**Disjointedness**				
VIS	0.002 (3.87e-04)	0.002 (3.96e-04)	0.873	0.931
SM	0.002 (3.44e-04)	0.002 (3.43e-04)	0.348	0.645
DAN	0.002 (3.16e-04)	0.002 (3.64e-04)	0.084	0.434
SAL	0.002 (3.46e-04)	0.002 (3.81e-04)	0.101	0.434
LIMB	0.003 (0.001)	0.003 (0.001)	0.135	0.434
CONT	0.002 (3.89e-04)	0.002 (3.61e-04)	0.172	0.5
DMN	0.002 (3.44e-04)	0.002 (3.17e-04)	0.040	0.32
SUB	0.003 (6.73e-04)	0.003 (4.8e-04)	0.716	0.848
**Cohesion**				
VIS	0.152 (0.014)	0.149 (0.016)	0.943	0.973
SM	0.157 (0.013)	0.159 (0.016)	0.576	0.802
DAN	0.15 (0.015)	0.148 (0.014)	0.263	0.562
SAL	0.156 (0.013)	0.154 (0.013)	0.666	0.848
LIMB	0.166 (0.012)	0.164 (0.014)	0.395	0.645
CONT	0.152 (0.013)	0.147 (0.015)	0.132	0.434
DMN	0.148 (0.016)	0.141 (0.016)	< 0.001	**0.004**
SUB	0.169 (0.012)	0.167 (0.012)	0.403	0.645

Supplementary Table 3 Significant associations between striatal dopamine transporter availability and dynamic network reconfiguration measures. We have included only the significant associations after FDR correction out of 576 comparisons. The results are divided into different sliding window lengths (SW15, SW19, SW23) in each diagnostic group. SW = Sliding Window, PD = Parkinson's disease, HC = Healthy Controls, VIS = Visual, SM = Somatomotor, DAN = Dorsal-Attention, SAL = Salient-Ventral Attention, LIMB = Limbic, CONT = Control, DMN = Default-mode, SUB = Subcorticals.

SW	Diagnosis	Network dynamic functional connectivity measure	Striatal dopamine transporter availability measure	Estimate (Std. error)	FDR-Corrected p-value
15	PD	LIMB Flexibility	Striatum Average	7.89e-03 (2.41e-03)	0.02
		LIMB Cohesion	Striatum Average	7.88e-03 (2.42e-03)	0.02
		LIMB Flexibility	Caudate Average	5.73e-03 (1.78e-03)	0.03
		LIMB Cohesion	Caudate Average	5.74e-03 (1.79e-03)	0.03
	HC	SAL Flexibility	Striatum Average	1.39e-02 (2.93e-03)	0.01
		SAL Promiscuity	Striatum Average	3.67e-02 (9.56e-03)	0.01
		CONT Promiscuity	Striatum Average	4.01e-02 (1.02e-02)	0.01
		SAL Cohesion	Striatum Average	1.38e-02 (2.97e-03)	0.01
		SM Flexibility	Caudate Average	1.58e-02 (4.57e-03)	0.02
		SAL Flexibility	Caudate Average	1.27e-02 (2.77e-03)	0.01
		LIMB Flexibility	Caudate Average	2.14e-02 (7.60e-03)	0.05
		CONT Flexibility	Caudate Average	1.67e-02 (4.73e-03)	0.02
		CONT Promiscuity	Caudate Average	4.17e-02 (1.37e-02)	0.03
		SM Cohesion	Caudate Average	1.57e-02 (4.47e-03)	0.02
		SAL Cohesion	Caudate Average	1.27e-02 (2.79e-03)	0.01

		CONT Cohesion	Caudate Average	1.67e-02 (4.72e-03)	0.02
		SUB Cohesion	Caudate Average	1.28e-02 (4.20e-03)	0.03
		SAL Flexibility	Putamen Average	1.44e-02 (3.21e-03)	0.01
		DAN Promiscuity	Putamen Average	3.76e-02 (1.15e-02)	0.04
		SAL Promiscuity	Putamen Average	4.18e-02 (1.02e-02)	0.01
		CONT Promiscuity	Putamen Average	4.59e-02 (1.37e-02)	0.04
		SAL Cohesion	Putamen Average	1.42e-02 (3.21e-03)	0.01
19	PD	LIMB Flexibility	Striatum Average	7.50e-03 (2.43e-03)	0.03
		VIS Promiscuity	Striatum Average	-2.12e-02 (6.17e-03)	0.03
		LIMB Cohesion	Striatum Average	7.47e-03 (2.44e-03)	0.03
	HC	SM Flexibility	Striatum Average	1.88e-02 (4.96e-03)	0.01
		SAL Flexibility	Striatum Average	1.34e-02 (2.55e-03)	< 0.01
		LIMB Flexibility	Striatum Average	2.70e-02 (9.62e-03)	0.04
		DMN Flexibility	Striatum Average	1.30e-02 (4.48e-03)	0.04
		CONT Promiscuity	Striatum Average	4.86e-02 (1.61e-02)	0.04
		SUB Promiscuity	Striatum Average	5.31e-02 (1.12e-02)	< 0.01
		SM Cohesion	Striatum Average	1.87e-02 (4.86e-03)	0.01
		SAL Cohesion	Striatum Average	1.32e-02 (2.60e-03)	< 0.01
		DMN Cohesion	Striatum Average	1.29e-02 (4.40e-03)	0.04
		SAL Disjointedness	Striatum Average	7.71e-04 (2.65e-04)	0.04
		SM Flexibility	Caudate Average	1.94e-02 (3.71e-03)	< 0.01
		SAL Flexibility	Caudate Average	1.21e-02 (2.44e-03)	< 0.01

		LIMB Flexibility	Caudate Average	2.06e-02 (7.16e-03)	0.05
		DMN Flexibility	Caudate Average	1.21e-02 (4.16e-03)	0.05
		SM Cohesion	Caudate Average	1.63e-02 (4.71e-03)	0.03
		SAL Cohesion	Caudate Average	1.20e-02 (2.50e-03)	< 0.01
		LIMB Cohesion	Caudate Average	2.06e-02 (7.00e-03)	0.05
		DMN Cohesion	Caudate Average	1.19e-02 (4.10e-03)	0.05
		SAL Flexibility	Putamen Average	1.33e-02 (2.74e-03)	0.01
		SAL Promiscuity	Putamen Average	5.98e-02 (1.31e-02)	0.01
		CONT Promiscuity	Putamen Average	5.59e-02 (1.74e-02)	0.03
		SUB Promiscuity	Putamen Average	6.22e-02 (1.49e-02)	0.01
		SAL Cohesion	Putamen Average	1.31e-02 (2.80e-03)	0.01
		SAL Disjointedness	Putamen Average	1.05e-03 (2.71e-04)	0.01
23	HC	SM Flexibility	Striatum Average	1.95e-02 (4.67e-03)	0.01
		SAL Flexibility	Striatum Average	1.33e-02 (2.19e-03)	< 0.01
		SM Cohesion	Striatum Average	1.94e-02 (4.55e-03)	0.01
		SAL Cohesion	Striatum Average	1.30e-02 (2.26e-03)	< 0.01
		SM Flexibility	Caudate Average	2.00e-02 (3.36e-03)	< 0.01
		SAL Flexibility	Caudate Average	1.26e-02 (2.70e-03)	< 0.01
		SM Cohesion	Caudate Average	1.99e-02 (3.26e-03)	< 0.01
		SAL Cohesion	Caudate Average	1.24e-02 (2.75e-03)	< 0.01
		SAL Flexibility	Putamen Average	1.32e-02 (2.42e-03)	< 0.01
		CONT Promiscuity	Putamen Average	4.61e-02 (1.43e-02)	0.05

		SAL Cohesion	Putamen Average	1.29e-02 (2.50e-03)	< 0.01
		SAL Disjointedness	Putamen Average	1.15e-03 (2.49e-04)	< 0.01

Supplementary Table 4 Significant associations between striatal dopamine transporter availability and nodal dynamic network reconfiguration measures in Parkinson's disease patients using Schaefer's parcellation. We have included only the significant associations, after FDR correction, from 30456 comparisons. No significant findings survived multiple-comparison correction for a sliding window length of 19 TRs. SW = Sliding Window.

SW	Nodal dynamic functional connectivity measure	Striatal dopamine transporter availability measure	Estimate (Std. error)	FDR-Corrected p-value
15	Right Temporoparietal junction 7 Disjointedness	Striatum Average	-1.56e-03 (3.59e-04)	0.046
	Right Temporoparietal junction 7 Disjointedness	Caudate Average	-1.20e-03 (2.73e-04)	0.043
	Right Temporoparietal junction 7 Disjointedness	Putamen Average	-1.94e-03 (4.41e-04)	0.04
23	Right Temporoparietal junction 7 Disjointedness	Caudate Average	-2.98e-02 (6.70e-03)	0.032

Supplementary Table 5 Significant associations between striatum average dopamine transporter availability and nodal dynamic network reconfiguration measures in Parkinson's disease patients using Glasser's parcellation. We have included only the significant associations, after FDR correction. SW = Sliding Window, Ig = Insular granular complex in the Glasser parcellation, H = Glasser cortical area H. Hippocampal body refers to the hippocampal body region from the Melbourne subcortical atlas.

SW	Nodal dynamic functional connectivity measure	Estimate (Std. error)	FDR-Corrected p-value
15	Right Ig flexibility	-1.45e-02 (3.44e-03)	0.037
	Right Ig cohesion	-1.47e-02 (3.26e-03)	0.024
19	Right Ig flexibility	-1.51e-02 (3.67e-03)	0.018
	Right Hippocampal body flexibility	1.53e-02 (3.58e-03)	0.015
	Left H promiscuity	3.72e-02 (8.41e-03)	0.015
	Right Hippocampal body promiscuity	3.88e-02 (8.98e-03)	0.015
	Right Ig cohesion	-1.57e-02 (3.73e-03)	0.015
	Right Hippocampal body cohesion	1.54e-02 (3.55e-03)	0.015
23	Left H promiscuity	3.85e-02 (9.19e-03)	0.040
	Right Hippocampal body promiscuity	4.56e-02 (9.21e-03)	0.003

Supplementary Table 6 Associations between MoCA scores and dynamic network reconfiguration measures in Parkinson's disease patients. The networks are: VIS = Visual, SM = Somatomotor, DAN = Dorsal-Attention, SAL = Salient-Ventral Attention, LIMB = Limbic, CONT = Control, DMN = Default-mode, SUB = Subcorticals.

SW	Dynamic network reconfiguration measure	Estimate (Std. error)	Uncorrected p-value	FDR-corrected p-value
19	**Flexibility**			
	VIS	22 (10)	0.031	0.121
	SM	17 (11)	0.123	0.197
	DAN	33 (12)	0.007	0.057
	SAL	26 (13)	0.045	0.121
	LIMB	-0.18 (12)	0.988	0.988
	CONT	21 (11)	0.076	0.151
	DMN	7.4 (11)	0.498	0.569
	SUB	19 (15)	0.195	0.260
	**Promiscuity**			
	VIS	10 (4.7)	0.033	0.266
	SM	3.5 (5.4)	0.519	0.807
	DAN	8.8 (5.4)	0.106	0.425
	SAL	4.6 (5.5)	0.404	0.807
	LIMB	-3 (5.9)	0.605	0.807
	CONT	3.6 (5.4)	0.500	0.807
	DMN	-1.2 (4.9)	0.813	0.920
	SUB	0.64 (6.4)	0.920	0.920
	**Disjointedness**			
	VIS	-65 (4.4e+02)	0.882	0.882
	SM	-1.5e+02 (5.3e+02)	0.782	0.882
	DAN	3.4e+02 (4.7e+02)	0.477	0.882
	SAL	-1.1e+03 (4.4e+02)	0.014	0.110
	LIMB	82 (1.5e+02)	0.576	0.882
	CONT	4.6e+02 (4.5e+02)	0.312	0.882
	DMN	-1.9e+02 (5.4e+02)	0.721	0.882

	SUB	-2.6e+02 (3.6e+02)	0.482	0.882
	**Cohesion**			
	VIS	22 (10)	0.030	0.105
	SM	17 (11)	0.120	0.192
	DAN	33 (12)	0.007	0.057
	SAL	27 (13)	0.039	0.105
	LIMB	-0.74 (12)	0.951	0.951
	CONT	20 (11)	0.080	0.160
	DMN	5.5 (11)	0.617	0.706
	SUB	20 (15)	0.184	0.245
15	**Flexibility**			
	VIS	23 (10)	0.027	0.071
	SM	19 (11)	0.096	0.153
	DAN	36 (12)	0.003	**0.017**
	SAL	37 (13)	0.004	**0.017**
	LIMB	11 (12)	0.378	0.432
	CONT	22 (12)	0.066	0.131
	DMN	8.2 (11)	0.458	0.458
	SUB	23 (15)	0.128	0.171
	**Promiscuity**			
	VIS	11 (4.7)	0.023	0.093
	SM	6 (5.6)	0.282	0.426
	DAN	13 (5.5)	0.022	0.093
	SAL	7 (5.4)	0.202	0.426
	LIMB	5.6 (6.1)	0.368	0.426
	CONT	5.9 (5.5)	0.284	0.426
	DMN	0.16 (5.1)	0.975	0.975
	SUB	6 (6.7)	0.372	0.426
	**Disjointedness**			
	VIS	30 (4.4e+02)	0.946	0.946
	SM	-1.2e+02 (5.3e+02)	0.827	0.945
	DAN	6e+02 (4.9e+02)	0.220	0.663
	SAL	-6.6e+02 (4.5e+02)	0.147	0.663

	LIMB	46 (1.4e+02)	0.748	0.945
	CONT	5.6e+02 (4.8e+02)	0.249	0.663
	DMN	-2.9e+02 (5.8e+02)	0.616	0.945
	SUB	-1.6e+02 (3.7e+02)	0.669	0.945
	**Cohesion**			
	VIS	23 (10)	0.026	0.069
	SM	19 (11)	0.093	0.148
	DAN	36 (12)	0.003	**0.014**
	SAL	38 (13)	0.003	**0.014**
	LIMB	10 (12)	0.398	0.447
	CONT	21 (12)	0.070	0.139
	DMN	8.4 (11)	0.447	0.447
	SUB	24 (15)	0.122	0.163
23	**Flexibility**			
	VIS	21 (10)	0.041	0.138
	SM	16 (11)	0.143	0.229
	DAN	31 (12)	0.012	0.095
	SAL	25 (13)	0.052	0.138
	LIMB	0.38 (12)	0.975	0.975
	CONT	21 (11)	0.070	0.140
	DMN	6.8 (11)	0.522	0.597
	SUB	18 (15)	0.221	0.295
	**Promiscuity**			
	VIS	9.4 (4.7)	0.049	0.391
	SM	1.7 (5.2)	0.746	0.852
	DAN	6.7 (5.2)	0.198	0.605
	SAL	4.2 (5.3)	0.428	0.605
	LIMB	-5.4 (5.6)	0.337	0.605
	CONT	3.9 (5.2)	0.454	0.605
	DMN	-3.7 (4.8)	0.434	0.605
	SUB	-0.94 (6.1)	0.876	0.876
	**Disjointedness**			
	VIS	-18 (4.3e+02)	0.966	0.966

	SM	-1e+02 (5.2e+02)	0.841	0.966
	DAN	1.4e+02 (4.7e+02)	0.765	0.966
	SAL	-9.3e+02 (4.4e+02)	0.035	0.282
	LIMB	1.5e+02 (1.5e+02)	0.302	0.805
	CONT	6e+02 (4.7e+02)	0.204	0.805
	DMN	-63 (5.3e+02)	0.905	0.966
	SUB	-2e+02 (3.5e+02)	0.568	0.966
	**Cohesion**			
	VIS	21 (10)	0.040	0.118
	SM	16 (11)	0.140	0.224
	DAN	31 (12)	0.012	0.093
	SAL	26 (13)	0.044	0.118
	LIMB	-0.6 (12)	0.960	0.960
	CONT	20 (11)	0.075	0.150
	DMN	7 (11)	0.516	0.589
	SUB	18 (14)	0.211	0.281

Supplementary Table 7 Baseline and one-year dynamic network reconfiguration measures based on the sliding window length of 15 TRs for 29 patients with the follow-up data. Fields with † had to be tested with the Wilcoxon rank sum test, since their differences did not follow a normal distribution. The networks are: VIS = Visual, SM = Somatomotor, DAN = Dorsal-Attention, SAL = Salient-Ventral Attention, LIMB = Limbic, CONT = Control, DMN = Default-mode, SUB = Subcorticals.

	**Baseline**	**Year 1**	**FDR-corrected p-value**
**Whole-brain average measures**			
Flexibility	0.155 (0.013)	0.159 (0.012)	**0.043***
Promiscuity	0.558 (0.023)	0.553 (0.025)	0.399
Disjointedness	2.34e-03 (1.67e-04)	2.24e-03 (2.14e-04)	0.074
Cohesion	0.152 (0.013)	0.157 (0.012)	**0.043***
**Network-average**			
**Flexibility**			
VIS	0.156 (0.016)	0.156 (0.016)	0.947
SM	0.164 (0.016)	0.167 (0.013)	0.307
DAN	0.15 (0.014)	0.155 (0.015)	0.139
SAL	0.156 (0.013)	0.16 (0.015)	0.09
LIMB	0.165 (0.015)	0.172 (0.012)	**0.044***
CONT	0.147 (0.016)	0.153 (0.013)	**0.044***
DMN†	0.146 [0.128, 0.154]	0.148 [0.137, 0.158]	**0.024***
SUB	0.172 (0.012)	0.177 (0.011)	**0.044***
**Promiscuity**			
VIS	0.563 (0.04)	0.551 (0.037)	0.494
SM	0.579 (0.031)	0.568 (0.036)	0.494
DAN	0.552 (0.024)	0.544 (0.033)	0.526
SAL	0.561 (0.027)	0.555 (0.034)	0.526
LIMB	0.576 (0.026)	0.574 (0.025)	0.883
CONT	0.54 (0.03)	0.541 (0.023)	0.896
DMN	0.526 (0.038)	0.533 (0.029)	0.526
SUB	0.592 (0.025)	0.585 (0.025)	0.526
**Disjointedness**			
VIS	2.36e-03 (3.96e-04)	2.16e-03 (3.84e-04)	0.101
SM	2.40e-03 (3.49e-04)	2.22e-03 (3.40e-04)	0.101
DAN	2.03e-03 (4.17e-04)	2.12e-03 (3.57e-04)	0.599
SAL†	2.05e-03 [1.81e-03, 2.29e-03]	1.98e-03 [1.73e-03, 2.20e-03]	0.717
LIMB	3.06e-03 (9.67e-04)	2.93e-03 (7.75e-04)	0.659
CONT	2.40e-03 (4.07e-04)	2.14e-03 (3.57e-04)	0.063
DMN	1.99e-03 (2.88e-04)	1.93e-03 (2.94e-04)	0.599
SUB	3.29e-03 (4.96e-04)	3.17e-03 (5.33e-04)	0.599
**Cohesion**			
VIS	0.153 (0.016)	0.154 (0.016)	0.901
SM	0.162 (0.016)	0.164 (0.013)	0.26
DAN	0.148 (0.014)	0.153 (0.015)	0.144
SAL	0.154 (0.013)	0.158 (0.014)	0.08
LIMB	0.162 (0.015)	0.169 (0.012)	**0.037***
CONT	0.145 (0.016)	0.151 (0.013)	**0.037***

DMN†	0.144 [0.126, 0.152]	0.147 [0.135, 0.156]	**0.024***
SUB	0.169 (0.012)	0.174 (0.011)	**0.037***

Supplementary Table 8 Baseline and one-year visit dynamic network reconfiguration measures based on the sliding window length of 23 TRs for 29 patients with the follow-up data. Fields with † had to be tested with the Wilcoxon rank sum test since their differences did not follow a normal distribution. The networks are: VIS = Visual, SM = Somatomotor, DAN = Dorsal-Attention, SAL = Salient-Ventral Attention, LIMB = Limbic, CONT = Control, DMN = Default-mode, SUB = Subcorticals.

	**Baseline**	**Year 1**	**FDR-corrected p-value**
**Whole-brain average measures**			
Flexibility	0.151 (0.014)	0.156 (0.012)	**0.045***
Promiscuity	0.561 (0.025)	0.553 (0.026)	0.204
Disjointedness	2.37e-03 (1.63e-04)	2.26e-03 (2.06e-04)	**0.045***
Cohesion	0.149 (0.014)	0.153 (0.012)	**0.045***
**Network-average**			
**Flexibility**			
VIS	0.152 (0.016)	0.152 (0.016)	0.906
SM	0.161 (0.017)	0.163 (0.013)	0.413
DAN	0.147 (0.014)	0.151 (0.015)	0.175
SAL	0.152 (0.014)	0.157 (0.015)	0.075
LIMB	0.162 (0.015)	0.168 (0.012)	**0.05***
CONT	0.144 (0.016)	0.149 (0.013)	**0.05***
DMN†	0.143 [0.123, 0.15]	0.146 [0.134, 0.155]	**0.013***
SUB	0.169 (0.013)	0.174 (0.011)	**0.05***
**Promiscuity**			
VIS†	0.568 [0.555, 0.589]	0.565 [0.522, 0.578]	0.086
SM	0.584 (0.035)	0.567 (0.036)	0.086
DAN	0.556 (0.027)	0.542 (0.033)	0.18
SAL	0.565 (0.029)	0.555 (0.037)	0.298
LIMB	0.579 (0.028)	0.574 (0.028)	0.573
CONT	0.543 (0.03)	0.538 (0.026)	0.573
DMN	0.528 (0.039)	0.532 (0.031)	0.658
SUB	0.596 (0.028)	0.586 (0.026)	0.298
**Disjointedness**			
VIS	2.41e-03 (4.27e-04)	2.22e-03 (4.04e-04)	0.175
SM	2.40e-03 (2.98e-04)	2.21e-03 (3.17e-04)	0.115
DAN	2.05e-03 (4.33e-04)	2.10e-03 (4.28e-04)	0.579
SAL†	1.93e-03 [1.71e-03, 2.38e-03]	2.10e-03 [1.87e-03, 2.26e-03]	0.579
LIMB†	3.33e-03 [2.57e-03, 4.04e-03]	2.96e-03 [2.44e-03, 3.90e-03]	0.474
CONT	2.38e-03 (3.60e-04)	2.21e-03 (3.80e-04)	0.175
DMN	2.01e-03 (3.26e-04)	1.93e-03 (3.29e-04)	0.474
SUB	3.38e-03 (4.95e-04)	3.13e-03 (4.57e-04)	0.115
**Cohesion**			
VIS	0.15 (0.016)	0.15 (0.015)	0.864
SM	0.158 (0.017)	0.161 (0.013)	0.356
DAN	0.145 (0.014)	0.149 (0.015)	0.177
SAL	0.15 (0.014)	0.155 (0.015)	0.068

LIMB	0.159 (0.015)	0.165 (0.012)	**0.037***
CONT	0.141 (0.016)	0.147 (0.013)	**0.037***
DMN†	0.141 [0.121, 0.148]	0.144 [0.132, 0.154]	**0.01***
SUB	0.166 (0.013)	0.17 (0.011)	**0.037***

Supplementary Table 9 Bayesian Latent Change Score model results. The model parameters fitted to the longitudinal data using the "*blavaan*" package are shown, with the changes from baseline to the year-one visit in the average striatal DaTscan binding ratios associated with changes in dynamic network reconfiguration measures for different sliding window lengths (SW). The results here are limited to significant associations. PPP values in the range 0.05-0.95 indicate a good fit, with PPP = 0.5 corresponding to an excellent fit. BRMSEA values <0.05 show an excellent fit, while <0.08 indicate a good fit. BGH, adjusted BGH, and BMc are in the range of 0 - 1, with values closer to 0 indicating a poor fit, while values closer to 1 are indicative of a better fit. The significant findings that have acceptable fit measures are highlighted. Fit measures: PPP = Posterior Predictive P-values, BRMSEA = Bayesian Root Mean Square Error of Approximation, BGH = Bayesian Gamma Hat, BMc = Bayesian McDonald's Noncentrality Index. Networks: VIS = Visual, SM = Somatomotor, DAN = Dorsal Attention, SAL = Salient Ventral Attention, LIMB = Limbic, CONT = Control, DMN = Default mode, SUB = Subcorticals.

SW	Striatal Dopamine Transporter Availability Measure	Dynamic network reconfiguration measures	rho [95%CI]	PPP	BRMSEA	BGH	Adjusted BGH	BMc
19	Striatum Average	Whole-brain average Cohesion	-0.339 [-0.676, -0.003]	0.60	0.06	0.97	0.92	0.97
		Whole-brain average Flexibility	-0.347 [-0.681, -0.012]	0.61	0.06	0.98	0.92	0.97
		VIS Flexibility	-0.465 [-0.769, -0.160]	0.58	0.08	0.97	0.88	0.96
		DAN Flexibility	-0.410 [-0.733, -0.087]	0.57	0.07	0.97	0.90	0.96
		VIS Cohesion	-0.458 [-0.762, -0.155]	0.58	0.07	0.97	0.89	0.96
		DAN Cohesion	-0.409 [-0.728, -0.091]	0.59	0.07	0.97	0.91	0.96
		SAL Disjointedness	-0.347 [-0.687, -0.007]	0.62	0.06	0.98	0.92	0.97
	Caudate Average	Whole-brain average Cohesion	-0.423 [-0.745, -0.101]	0.62	0.06	0.97	0.92	0.97
		Whole-brain average Flexibility	-0.427 [-0.745, -0.109]	0.61	0.06	0.98	0.93	0.97

		VIS Flexibility	-0.550 [-0.825, -0.275]	0.60	0.07	0.97	0.91	0.97
		DAN Flexibility	-0.497 [-0.792, -0.202]	0.59	0.06	0.97	0.92	0.96
		SAL Flexibility	-0.347 [-0.688, -0.006]	0.63	0.06	0.98	0.92	0.97
		VIS Cohesion	-0.546 [-0.820, -0.271]	0.60	0.07	0.97	0.90	0.97
		DAN Cohesion	-0.496 [-0.786, -0.206]	0.60	0.06	0.97	0.92	0.97
	Putamen Average	SAL Disjointedness	-0.387 [-0.716, -0.059]	0.59	0.10	0.97	0.81	0.96
15	Striatum Average	Whole-brain average Cohesion	-0.358 [-0.693, -0.023]	0.56	0.07	0.97	0.90	0.96
		Whole-brain average Flexibility	-0.362 [-0.696, -0.028]	0.57	0.07	0.97	0.90	0.96
		VIS Flexibility	-0.476 [-0.780, -0.172]	0.54	0.09	0.97	0.87	0.96
		DAN Flexibility	-0.424 [-0.743, -0.104]	0.56	0.07	0.97	0.90	0.96
		VIS Cohesion	-0.473 [-0.775, -0.170]	0.55	0.08	0.97	0.88	0.96
		DAN Cohesion	-0.423 [-0.741, -0.106]	0.55	0.08	0.97	0.90	0.96
	Caudate Average	Whole-brain average Cohesion	-0.433 [-0.752, -0.114]	0.58	0.07	0.97	0.91	0.96
		Whole-brain average Flexibility	-0.433 [-0.753, -0.114]	0.59	0.06	0.97	0.92	0.96
		VIS Flexibility	-0.555 [-0.824, -0.286]	0.56	0.08	0.97	0.88	0.96
		DAN Flexibility	-0.503 [-0.793, -0.212]	0.57	0.07	0.97	0.91	0.96

		VIS Cohesion	-0.553 [-0.826, -0.280]	0.57	0.07	0.97	0.90	0.96
		DAN Cohesion	-0.504 [-0.797, -0.212]	0.56	0.07	0.97	0.91	0.96
23	Striatum Average	Whole-brain average Cohesion	-0.374 [-0.703, -0.044]	0.59	0.07	0.97	0.91	0.96
		Whole-brain average Flexibility	-0.380 [-0.708, -0.052]	0.59	0.07	0.97	0.91	0.96
		VIS Flexibility	-0.488 [-0.784, -0.191]	0.57	0.08	0.97	0.88	0.96
		DAN Flexibility	-0.394 [-0.721, -0.068]	0.55	0.08	0.97	0.89	0.96
		VIS Cohesion	-0.480 [-0.781, -0.180]	0.57	0.08	0.97	0.88	0.96
		DAN Cohesion	-0.396 [-0.720, -0.071]	0.56	0.08	0.97	0.90	0.96
		SAL Disjointedness	-0.392 [-0.721, -0.062]	0.61	0.06	0.98	0.91	0.97
	Caudate Average	Whole-brain average Cohesion	-0.448 [-0.761, -0.136]	0.61	0.06	0.97	0.92	0.97
		Whole-brain average Flexibility	-0.451 [-0.762, -0.140]	0.61	0.06	0.97	0.92	0.97
		VIS Flexibility	-0.566 [-0.834, -0.298]	0.60	0.07	0.97	0.90	0.97
		DAN Flexibility	-0.482 [-0.777, -0.187]	0.58	0.07	0.97	0.92	0.96
		SAL Flexibility	-0.367 [-0.696, -0.039]	0.63	0.06	0.98	0.93	0.97
		VIS Promiscuity	-0.360 [-0.696, -0.024]	0.61	0.09	0.98	0.81	0.97
		VIS Cohesion	-0.562 [-0.829, -0.295]	0.60	0.07	0.97	0.90	0.96

		DAN Cohesion	-0.483 [-0.780, -0.185]	0.57	0.07	0.97	0.91	0.96
		SAL Cohesion	-0.355 [-0.691, -0.020]	0.62	0.06	0.98	0.92	0.97
	Putamen Average	SAL Disjointedness	-0.437 [-0.750, -0.123]	0.59	0.10	0.97	0.81	0.96

Supplementary Table 10 Linear mixed effect model results for dynamic network reconfiguration measures as the dependent variable and the interaction between striatum average dopamine transporter availability and medication as the predictor variables. Time (baseline vs. year 1) was included as an additional fixed-effect, and the model was adjusted for age, sex, and baseline MDS-UPDRS part 3 scores, and accounting for repeated measures with a random intercept per subject.

SW	Dynamic network reconfiguration measures	Estimate (Std. error)	*p*-value	FDR-corrected *p*-value
19	**Whole-brain**			
	Flexibility	0.007 (0.007)	0.3555	0.5925
	Promiscuity	0.025 (0.02)	0.2218	0.5925
	Disjointedness	6.88e-05 (1.46e-04)	0.6406	0.6406
	Cohesion	0.007 (0.007)	0.3476	0.5925
	**Network Average**			
	**Flexibility**			
	VIS	2.66e-04 (0.012)	0.9826	0.9826
	SM	-6.61e-03 (0.009)	0.4628	0.7406
	DAN	0.009 (0.009)	0.3373	0.6746
	SAL	0.001 (0.008)	0.8983	0.9826
	LIMB	0.013 (0.009)	0.1691	0.6746
	CONT	0.01 (0.009)	0.2933	0.6746
	DMN	0.028 (0.009)	0.0044	**0.0353**
	SUB	5.75e-04 (0.007)	0.9307	0.9826
	**Promiscuity**			
	VIS	-1.08e-02 (0.033)	0.7452	0.9562
	SM	-4.74e-03 (0.026)	0.8558	0.9562
	DAN	0.033 (0.023)	0.1533	0.4089
	SAL	0.001 (0.026)	0.9562	0.9562
	LIMB	0.023 (0.025)	0.3601	0.7202
	CONT	0.044 (0.022)	0.0492	0.1967
	DMN	0.08 (0.028)	0.0057	**0.046**
	SUB	0.009 (0.022)	0.7032	0.9562
	**Disjointedness**			
	VIS	-4.28e-05 (3.44e-04)	0.9016	0.9949

	SM	-8.62e-05 (2.60e-04)	0.7417	0.9889
	DAN	1.23e-04 (3.17e-04)	0.6996	0.9889
	SAL	2.21e-06 (3.42e-04)	0.9949	0.9949
	LIMB	0.001 (7.50e-04)	0.0882	0.2351
	CONT	2.74e-04 (3.24e-04)	0.4002	0.8003
	DMN	5.88e-04 (2.55e-04)	0.0250	0.0998
	SUB	-9.82e-04 (3.38e-04)	0.0058	**0.0462**
	**Cohesion**			
	VIS	2.98e-04 (0.012)	0.9804	0.9804
	SM	-6.51e-03 (0.009)	0.4613	0.7381
	DAN	0.009 (0.009)	0.3317	0.6633
	SAL	0.001 (0.008)	0.8848	0.9804
	LIMB	0.011 (0.009)	0.2262	0.6633
	CONT	0.01 (0.009)	0.2834	0.6633
	DMN	0.027 (0.009)	0.0045	**0.0363**
	SUB	0.002 (0.007)	0.8179	0.9804
15	**Whole-brain**			
	Flexibility	0.008 (0.007)	0.2605	0.5714
	Promiscuity	0.017 (0.018)	0.3428	0.5714
	Disjointedness	4.52e-05 (1.53e-04)	0.7693	0.7693
	Cohesion	0.008 (0.007)	0.2487	0.5714
	**Network Average**			
	**Flexibility**			
	VIS	4.02e-04 (0.012)	0.9736	0.9736
	SM	-5.55e-03 (0.009)	0.5218	0.8348
	DAN	0.012 (0.009)	0.2086	0.5563
	SAL	0.003 (0.008)	0.7018	0.9357
	LIMB	0.01 (0.01)	0.2920	0.584
	CONT	0.012 (0.009)	0.1895	0.5563
	DMN	0.029 (0.009)	0.0038	**0.0302**
	SUB	0.001 (0.006)	0.8427	0.9631
	**Promiscuity**			
	VIS	-1.52e-02 (0.03)	0.6113	0.9528
	SM	-1.82e-02 (0.023)	0.4387	0.8775

	DAN	0.026 (0.023)	0.2468	0.6581
	SAL	-8.34e-03 (0.026)	0.7466	0.9528
	LIMB	-1.27e-03 (0.021)	0.9528	0.9528
	CONT	0.041 (0.021)	0.0552	0.2207
	DMN	0.066 (0.025)	0.0118	0.0947
	SUB	-2.16e-03 (0.02)	0.9165	0.9528
	**Disjointedness**			
	VIS	-8.86e-05 (2.78e-04)	0.7515	0.9731
	SM	6.50e-05 (2.83e-04)	0.8195	0.9731
	DAN	3.21e-05 (2.95e-04)	0.9137	0.9731
	SAL	-1.08e-05 (3.18e-04)	0.9731	0.9731
	LIMB	0.001 (7.10e-04)	0.0810	0.3238
	CONT	2.29e-04 (3.15e-04)	0.4709	0.9418
	DMN	3.29e-04 (2.38e-04)	0.1739	0.4638
	SUB	-1.15e-03 (4.00e-04)	0.0062	**0.0496**
	**Cohesion**			
	VIS	4.82e-04 (0.012)	0.9681	0.9681
	SM	-5.62e-03 (0.008)	0.5092	0.8005
	DAN	0.012 (0.009)	0.2003	0.534
	SAL	0.003 (0.008)	0.6837	0.8005
	LIMB	0.009 (0.01)	0.3770	0.754
	CONT	0.012 (0.009)	0.1798	0.534
	DMN	0.028 (0.009)	0.0037	**0.0293**
	SUB	0.002 (0.006)	0.7004	0.8005
23	**Whole-brain**			
	Flexibility	0.005 (0.007)	0.4427	0.5747
	Promiscuity	0.023 (0.02)	0.2651	0.5747
	Disjointedness	1.11e-04 (1.49e-04)	0.4597	0.5747
	Cohesion	0.005 (0.007)	0.4380	0.5747
	**Network Average**			
	**Flexibility**			
	VIS	-1.01e-04 (0.012)	0.9934	0.9934
	SM	-8.58e-03 (0.009)	0.3459	0.5535
	DAN	0.01 (0.009)	0.3050	0.5535

	SAL	-1.40e-03 (0.009)	0.8730	0.9934
	LIMB	0.013 (0.009)	0.1739	0.5535
	CONT	0.009 (0.009)	0.3251	0.5535
	DMN	0.027 (0.009)	0.0048	**0.0386**
	SUB	-1.19e-03 (0.007)	0.8590	0.9934
	**Promiscuity**			
	VIS	-1.23e-02 (0.033)	0.7076	0.9092
	SM	-9.08e-03 (0.025)	0.7151	0.9092
	DAN	0.039 (0.023)	0.1010	0.2692
	SAL	-7.17e-03 (0.028)	0.7956	0.9092
	LIMB	0.017 (0.024)	0.4740	0.9092
	CONT	0.045 (0.022)	0.0468	0.1873
	DMN	0.077 (0.027)	0.0064	0.0513
	SUB	8.99e-04 (0.023)	0.9689	0.9689
	**Disjointedness**			
	VIS	2.10e-05 (3.42e-04)	0.9514	0.9514
	SM	-1.18e-04 (2.60e-04)	0.6528	0.8704
	DAN	2.62e-04 (3.26e-04)	0.4265	0.853
	SAL	7.67e-05 (3.61e-04)	0.8325	0.9514
	LIMB	0.001 (7.52e-04)	0.0774	0.2065
	CONT	1.87e-04 (3.12e-04)	0.5522	0.8704
	DMN	5.74e-04 (2.65e-04)	0.0349	0.1397
	SUB	-9.26e-04 (3.52e-04)	0.0116	0.0931
	**Cohesion**			
	VIS	-1.43e-04 (0.012)	0.9905	0.9905
	SM	-8.51e-03 (0.009)	0.3413	0.5461
	DAN	0.01 (0.009)	0.3050	0.5461
	SAL	-1.34e-03 (0.009)	0.8774	0.9905
	LIMB	0.011 (0.009)	0.2413	0.5461
	CONT	0.009 (0.009)	0.3144	0.5461
	DMN	0.026 (0.009)	0.0049	**0.0391**
	SUB	-2.92e-04 (0.007)	0.9655	0.9905

Supplementary Figure 1 Significant group differences in nodal dynamic network reconfiguration measures between healthy controls and Parkinson's disease patients for sliding window length of 15 TRs using Schafer's parcellation. Colorbars show t-values. The blue range means lower and the red/yellow range indicates higher in patients compared to the controls. There were no significant differences in disjointedness in subcortical regions.

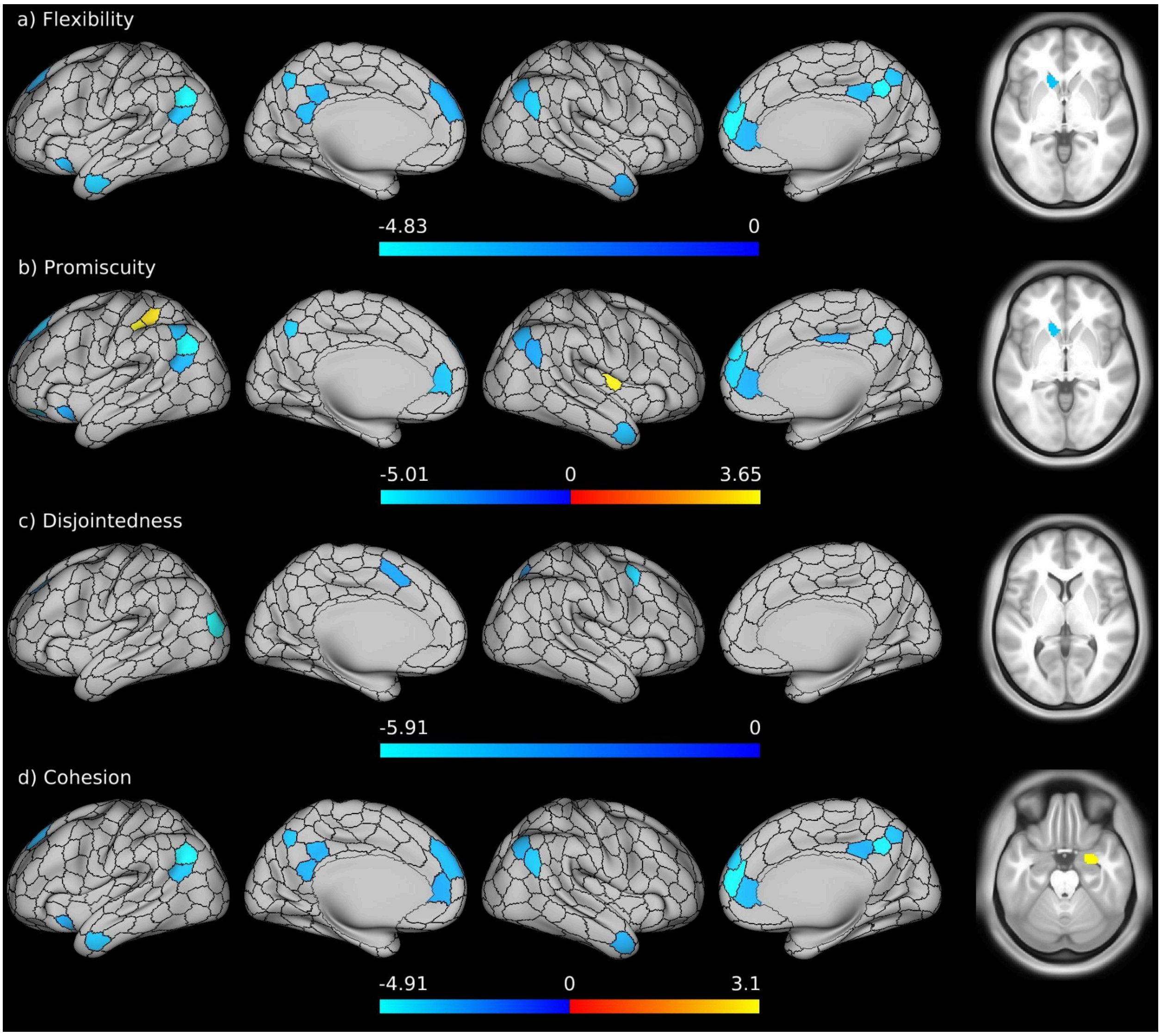

Supplementary Figure 2 Significant group differences in nodal dynamic network reconfiguration measures between healthy controls and Parkinson's disease patients for sliding window length of 23 TRs using Schafer's parcellation. Colorbars show t-values. The blue range means lower and the red/yellow range indicates higher in patients compared to the controls. There were no significant differences in flexibility, disjointedness, and cohesion in subcortical regions.

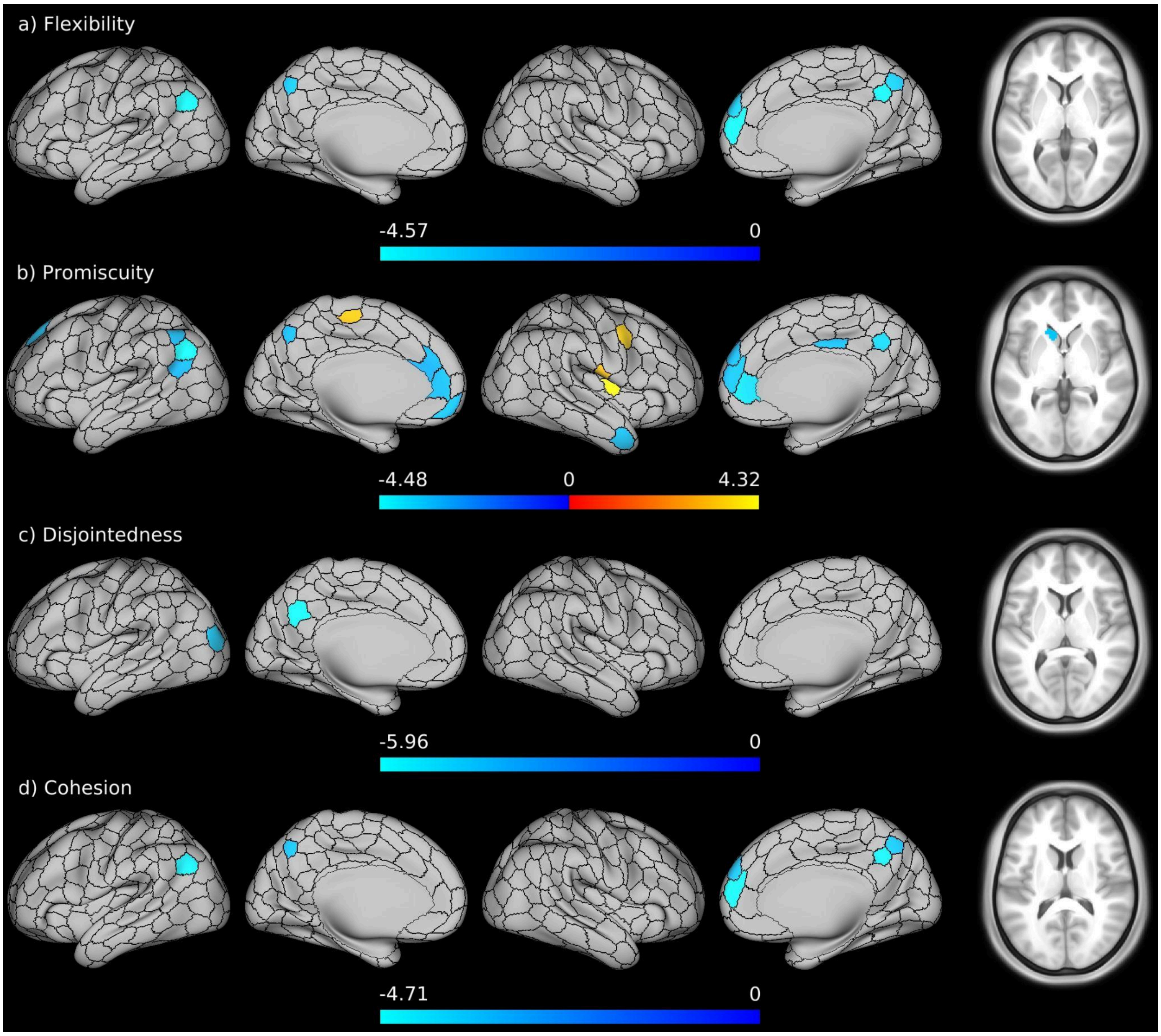

Supplementary Figure 3 Significant group differences in nodal dynamic network reconfiguration measures between healthy controls and Parkinson's disease patients for a sliding window length of 15 TRs, using Glasser's parcellation. Colorbars show t-values. The blue range indicates lower values, and the red/yellow range indicates higher values in patients compared to controls.

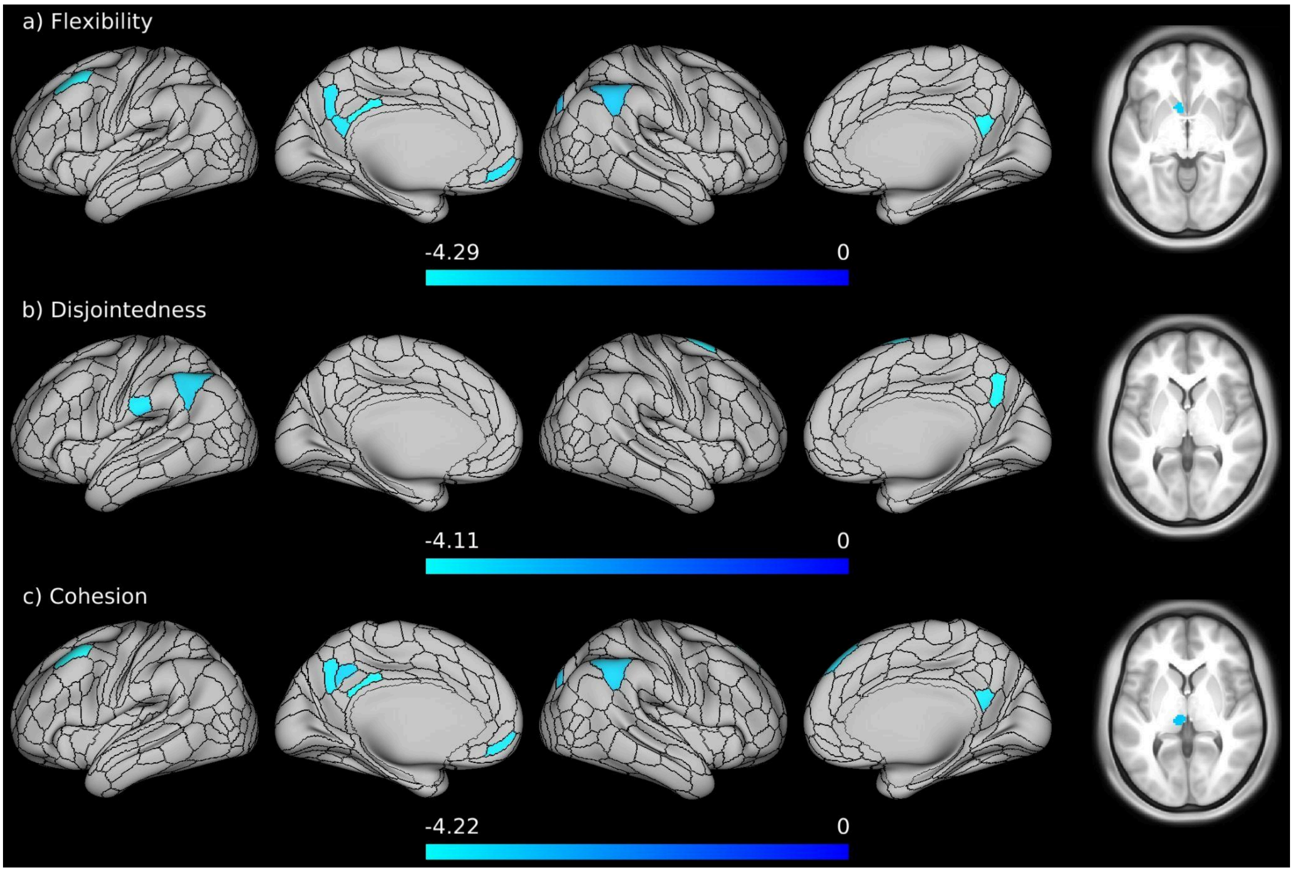

Supplementary Figure 4 Significant group differences in nodal dynamic network reconfiguration measures between healthy controls and Parkinson's disease patients for a sliding window length of 19 TRs, using Glasser's parcellation. Colorbars show t-values. The blue range indicates lower values, and the red/yellow range indicates higher values in patients compared to controls.

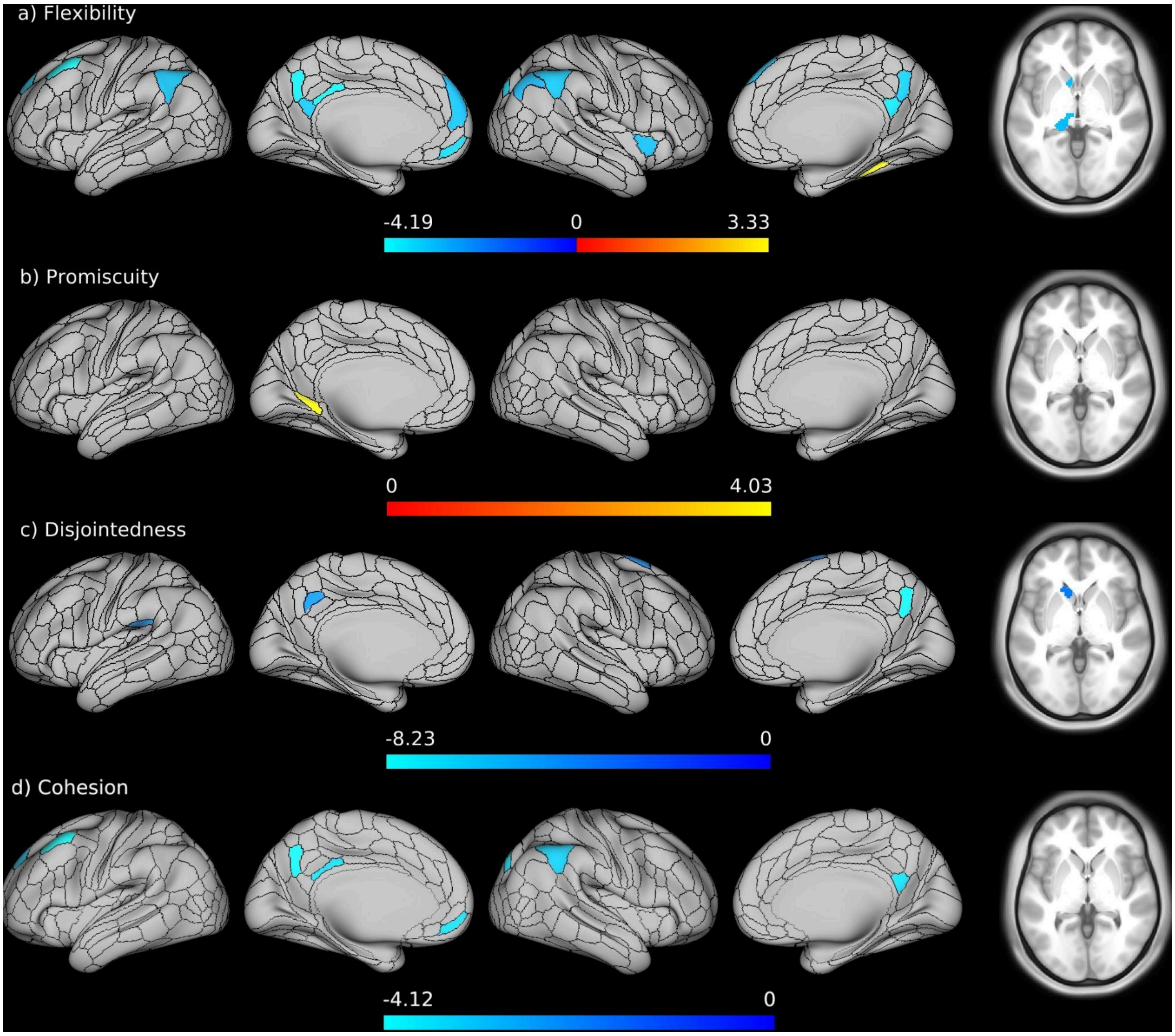

Supplementary Figure 5 Significant group differences in nodal dynamic network reconfiguration measures between healthy controls and Parkinson's disease patients for a sliding window length of 23 TRs, using Glasser's parcellation. Colorbars show t-values. The blue range indicates lower values, and the red/yellow range indicates higher values in patients compared to controls.

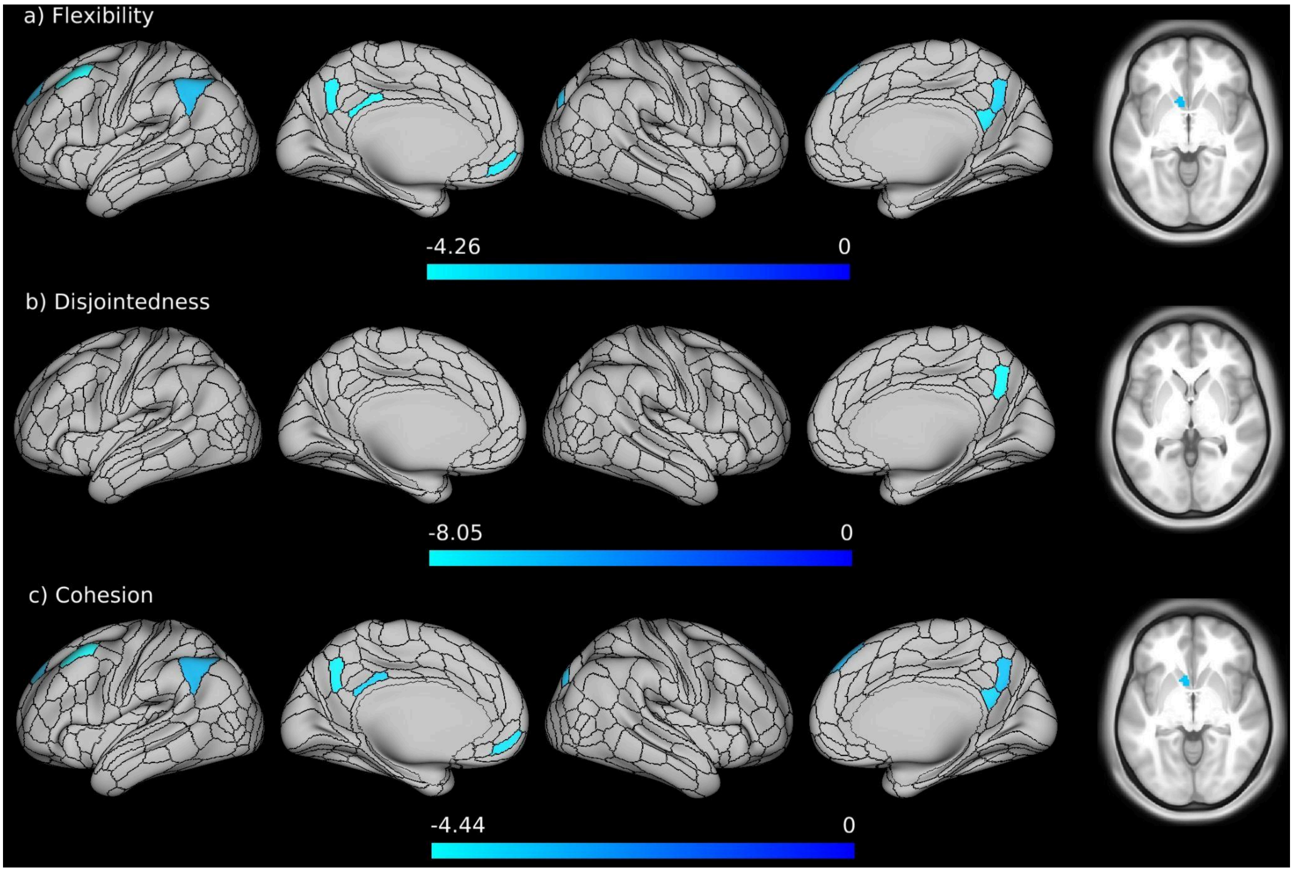

Supplementary Figure 6 Nodal dynamic network reconfiguration measures that were significantly associated with striatum average dopamine transporter availability for sliding window length of 19 TRs in healthy controls using Schaefer's parcellation. Colorbars show t-values.

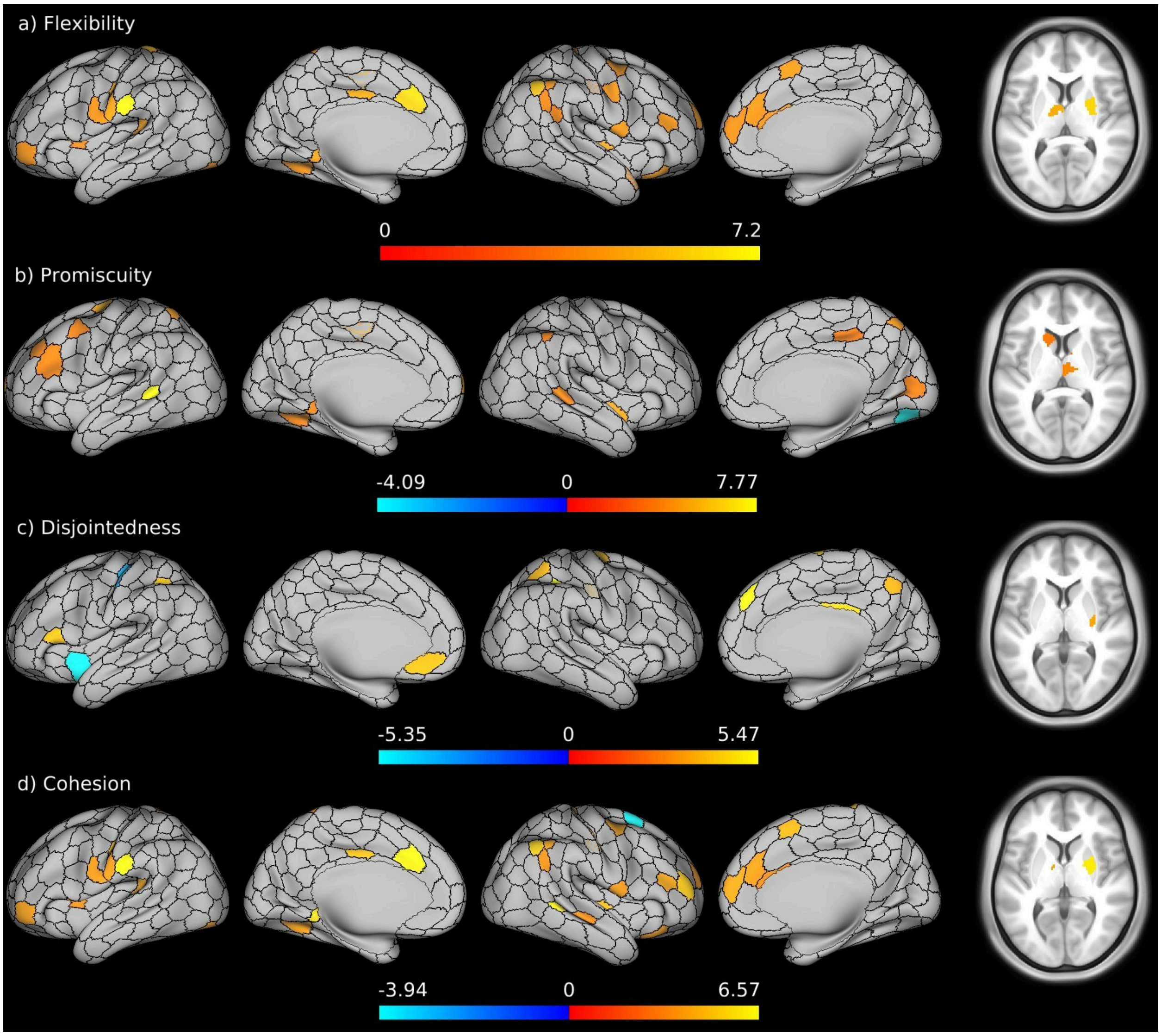

Supplementary Figure 7 Nodal dynamic network reconfiguration measures that were significantly associated with striatum average dopamine transporter availability for sliding window length of 15 TRs in healthy controls using Schaefer's parcellation. Colorbars show t-values.

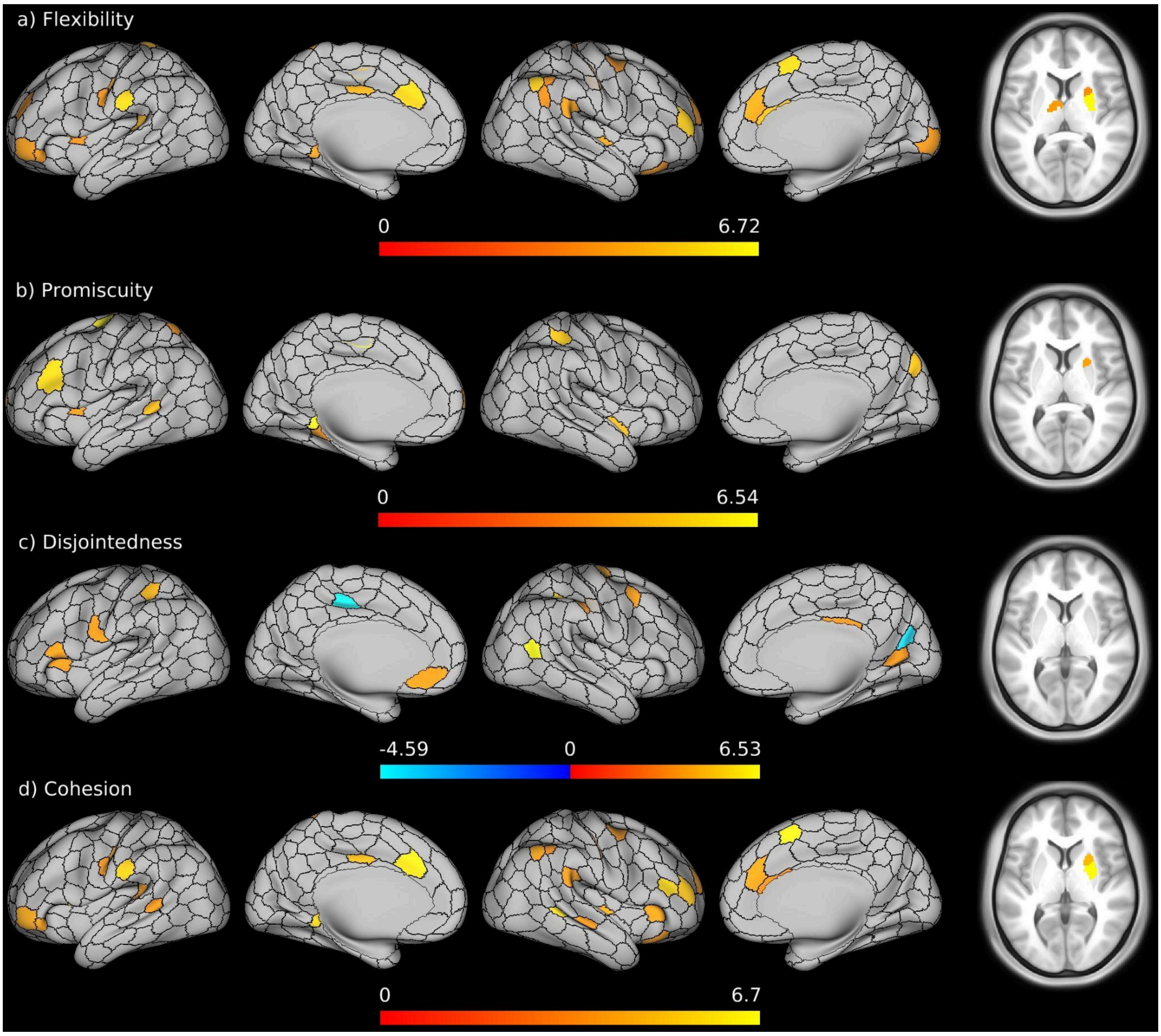

Supplementary Figure 8 Nodal dynamic network reconfiguration measures that were significantly associated with striatum average dopamine transporter availability for sliding window length of 23 TRs in healthy controls using Schaefer's parcellation. Colorbars show t-values.

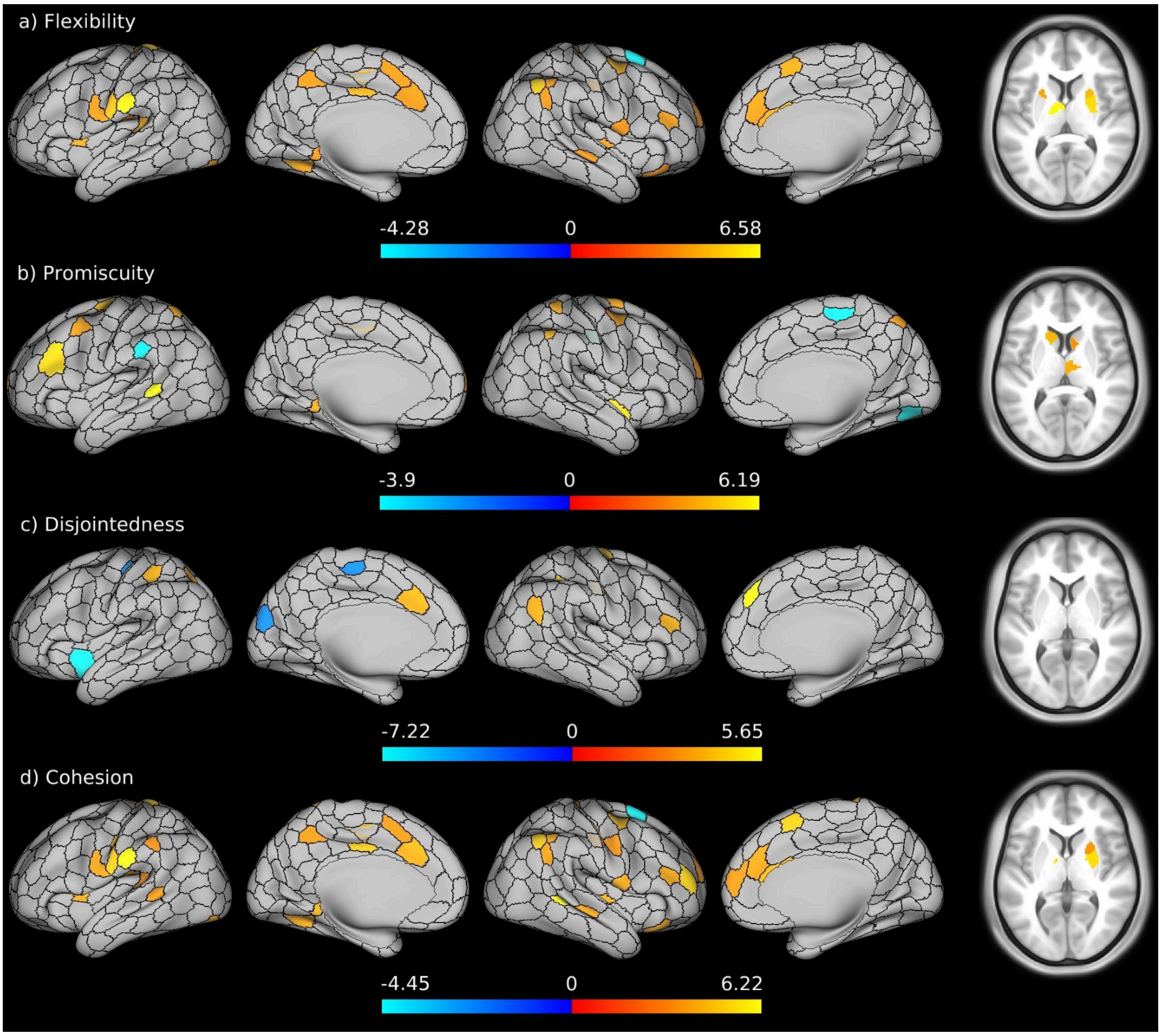

Supplementary Figure 9 Comparison of uncorrected significant findings ($p < 0.001$) across parcellation atlases. Results derived from the Glasser atlas are shown in red, those based on the Schaefer parcellation in blue, and their overlap in purple. Panels illustrate nodal associations for (a) striatum average dopamine transporter availability, (b) cognitive performance (MoCA), and (c) motor performance (MDS-UPDRS Part 3). Overall, several overlapping or anatomically proximate regions were identified across the two atlases for striatal dopamine transporter availability and cognitive performance, particularly for flexibility. In contrast, the spatial patterns of significant regions differed more substantially between atlases for motor performance. The results were based on the sliding window length of 19 TRs.

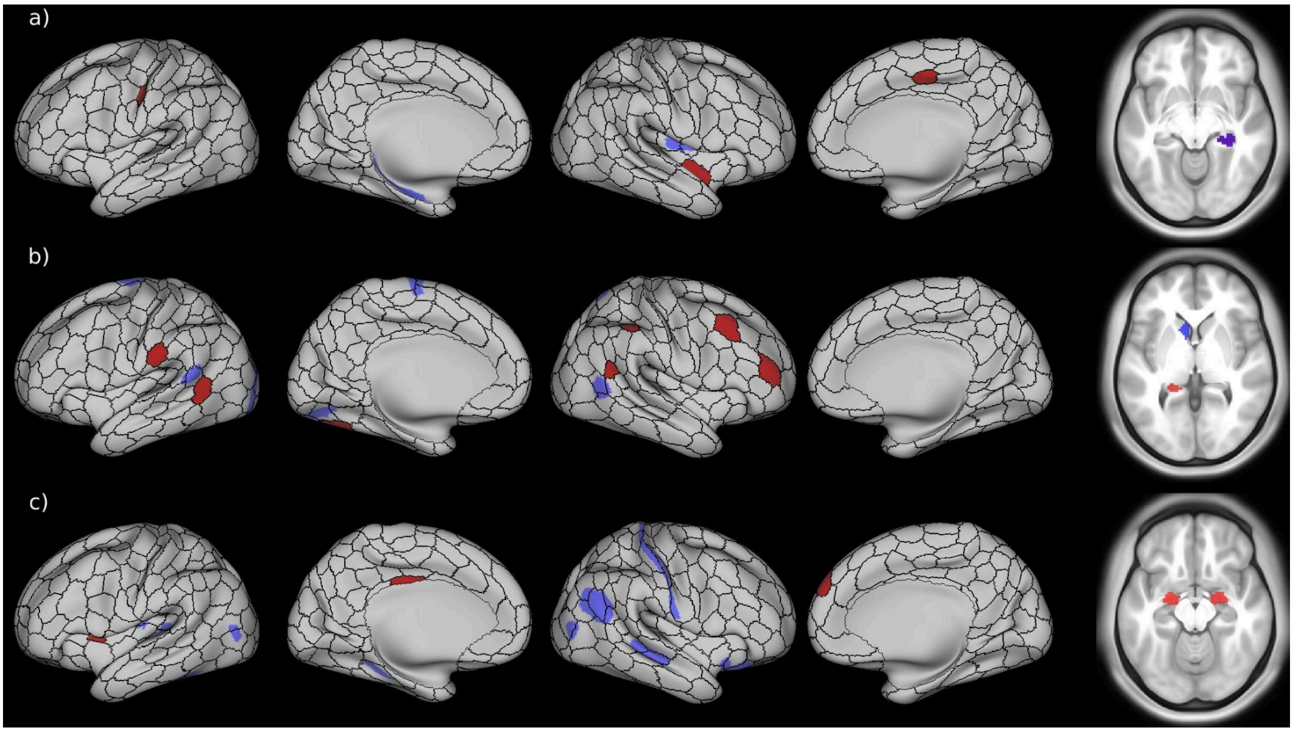

Supplementary Figure 10 Nodal dynamic network reconfiguration measures that were significantly associated with the total MoCA score for sliding window length of 15 TRs in Parkinson's disease patients using Schaefer's parcellation. No significant associations survived the multiple comparison correction for nodal disjointedness measures and subcortical regions. Colorbars show t-values.

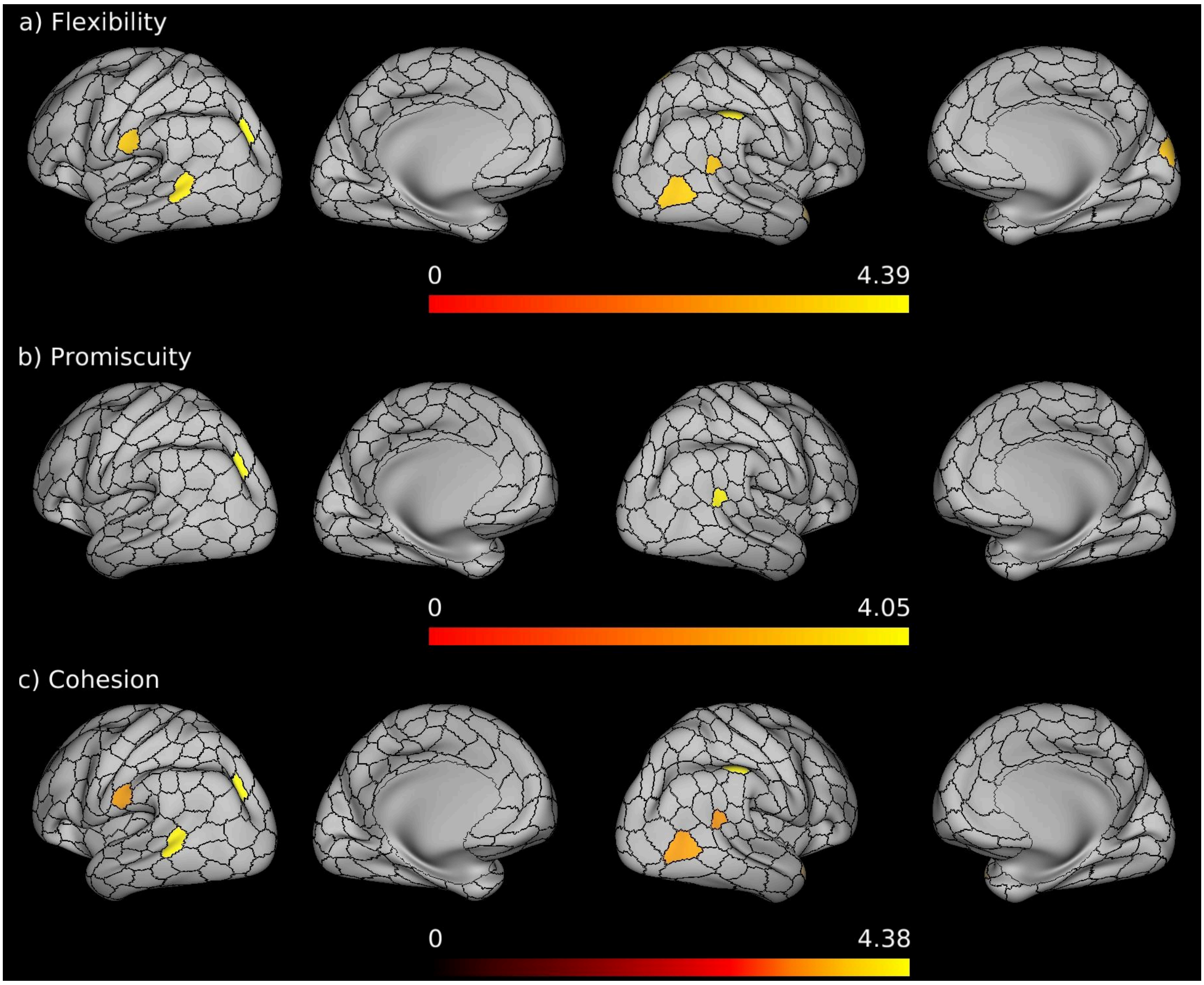

Supplementary Figure 11 Nodal dynamic network reconfiguration measures that were significantly associated with the total MoCA score for sliding window length of 23 TRs in Parkinson's disease patients using Schaefer's parcellation. No significant associations survived the multiple comparison correction for nodal promiscuity measures and subcortical structures. Colorbars show t-values.

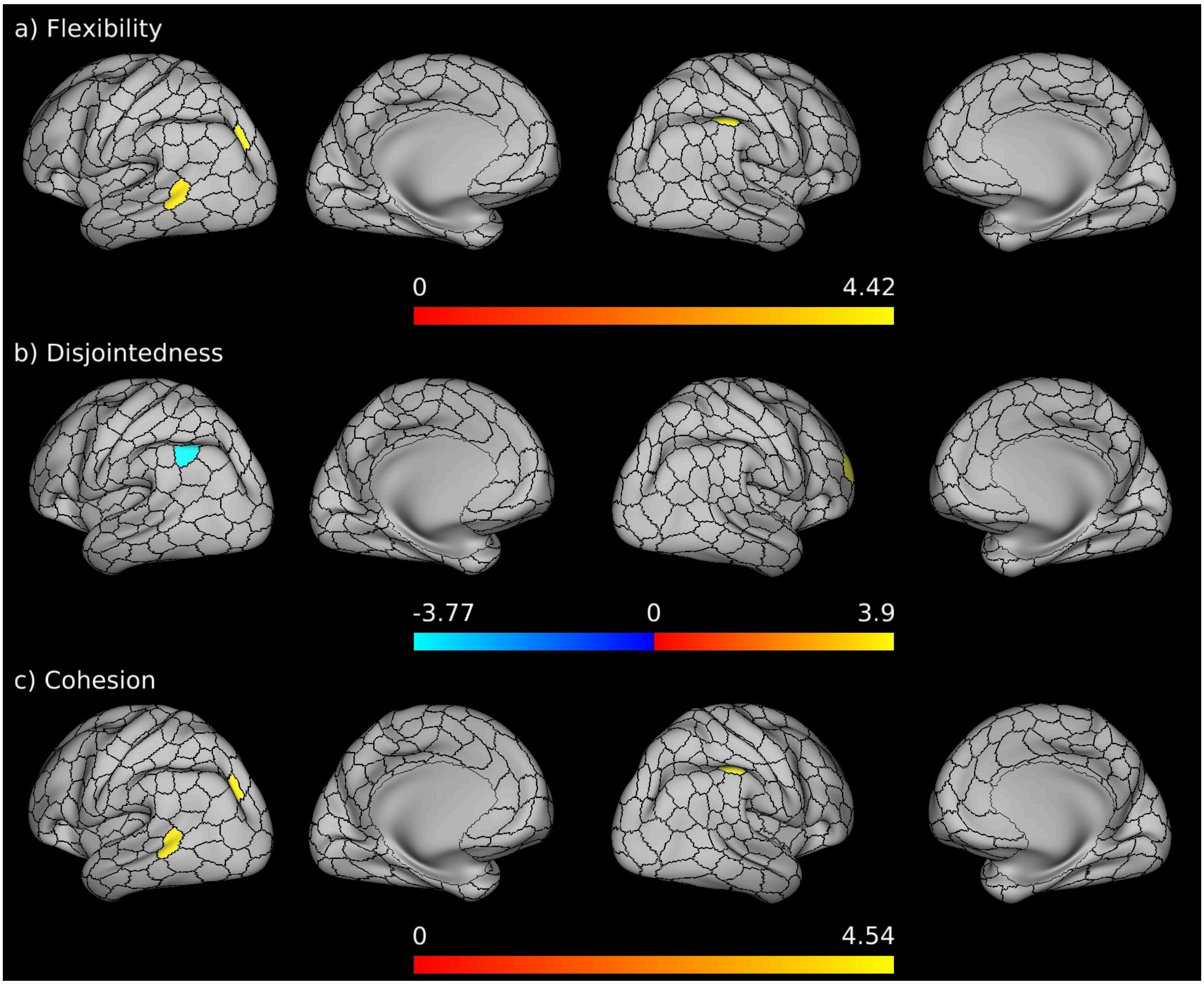

Supplementary Figure 12 Nodal dynamic network reconfiguration measures that were significantly associated with the total MoCA score for sliding window length of 15 TRs in Parkinson's disease patients, using Glasser's atlas. No significant associations survived the multiple comparison correction for nodal disjointedness and promiscuity measures. Colorbars show t-values.

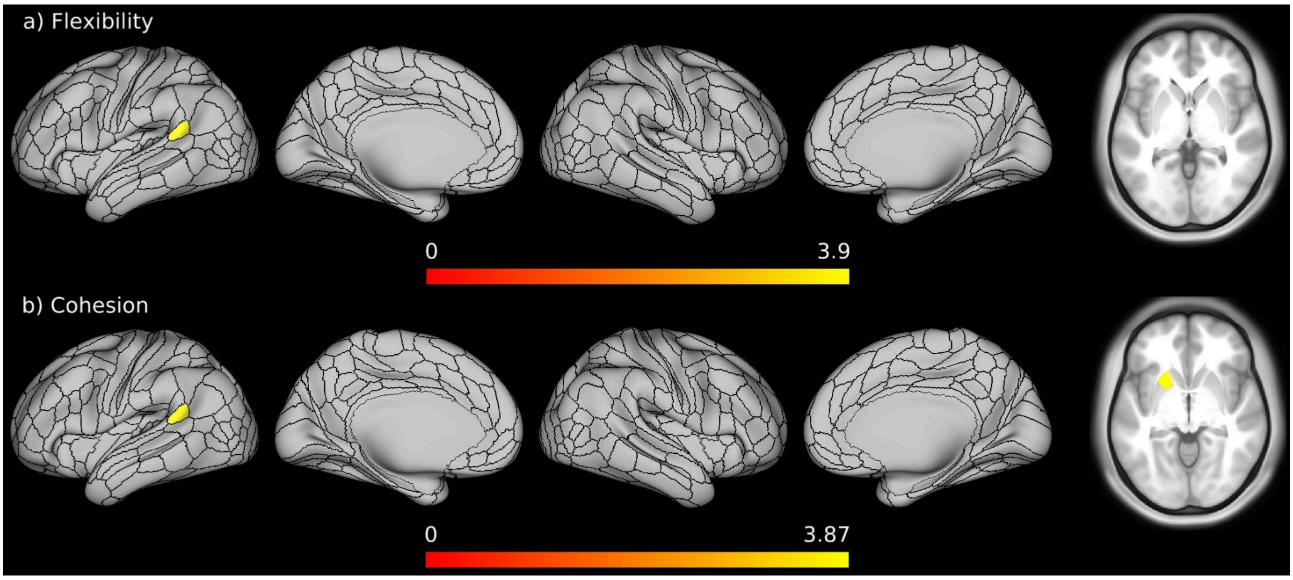

Supplementary Figure 13 Nodal dynamic network reconfiguration measures that were significantly associated with the total MoCA score for sliding window length of 23 TRs in Parkinson's disease patients, using Glasser's atlas. No significant associations survived the multiple comparison correction for nodal disjointedness and cohesion measures. Colorbars show t-values.

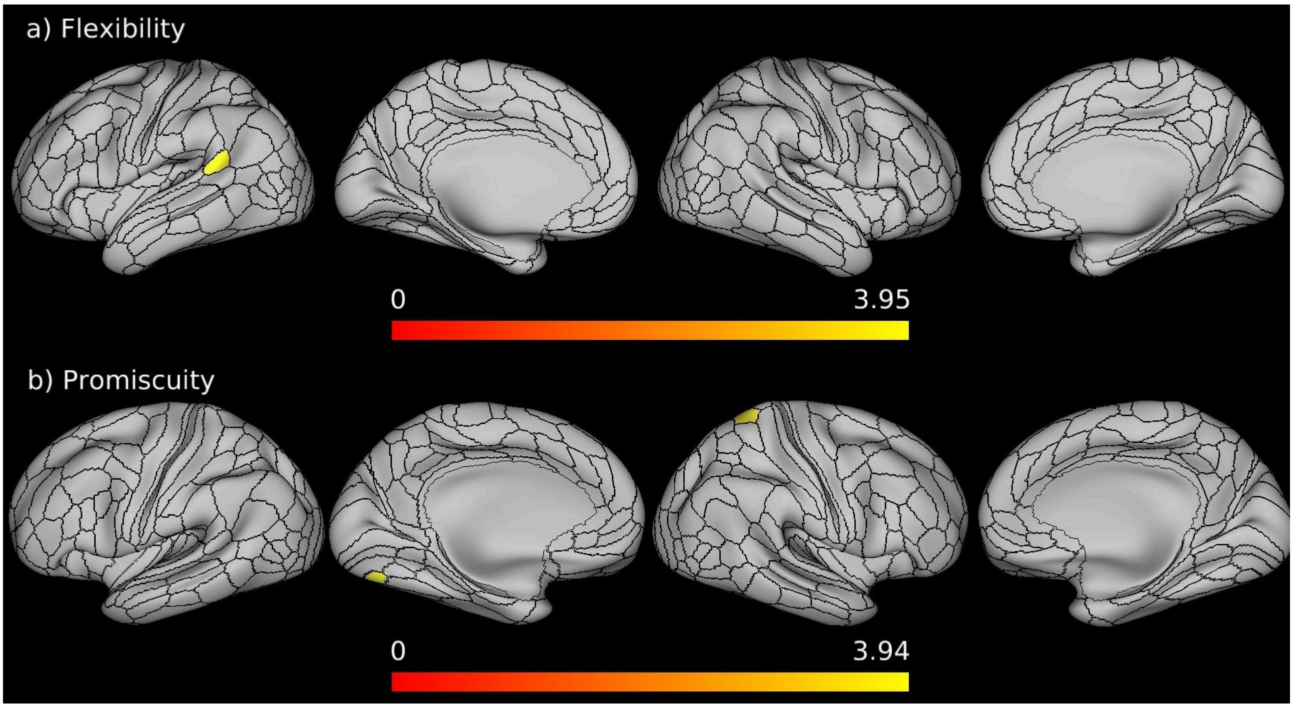

Supplementary Figure 14 Nodal dynamic network reconfiguration measures that were significantly associated with the MDS-UPDRS part 3 total score for sliding window length of 15 TRs in Parkinson's disease patients, using Schaefer's parcellation. Colorbars show t-values. There were no significant associations for promiscuity.

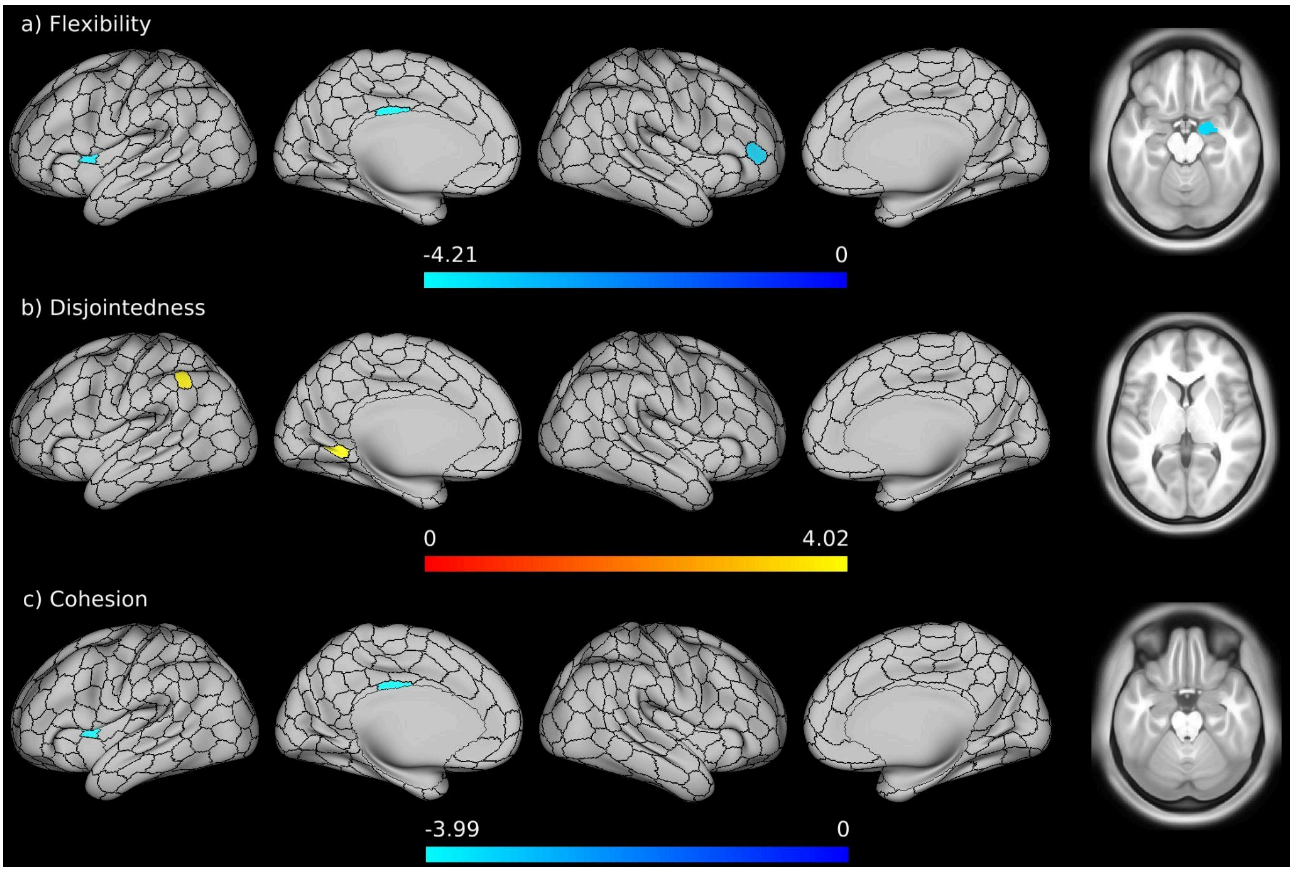

Supplementary Figure 15 Nodal dynamic network reconfiguration measures that were significantly associated with the MDS-UPDRS part 3 total score for sliding window length of 15 TRs in Parkinson's disease patients, using Glasser's atlas. Colorbars show t-values. There were no significant associations for promiscuity and subcortical regions.

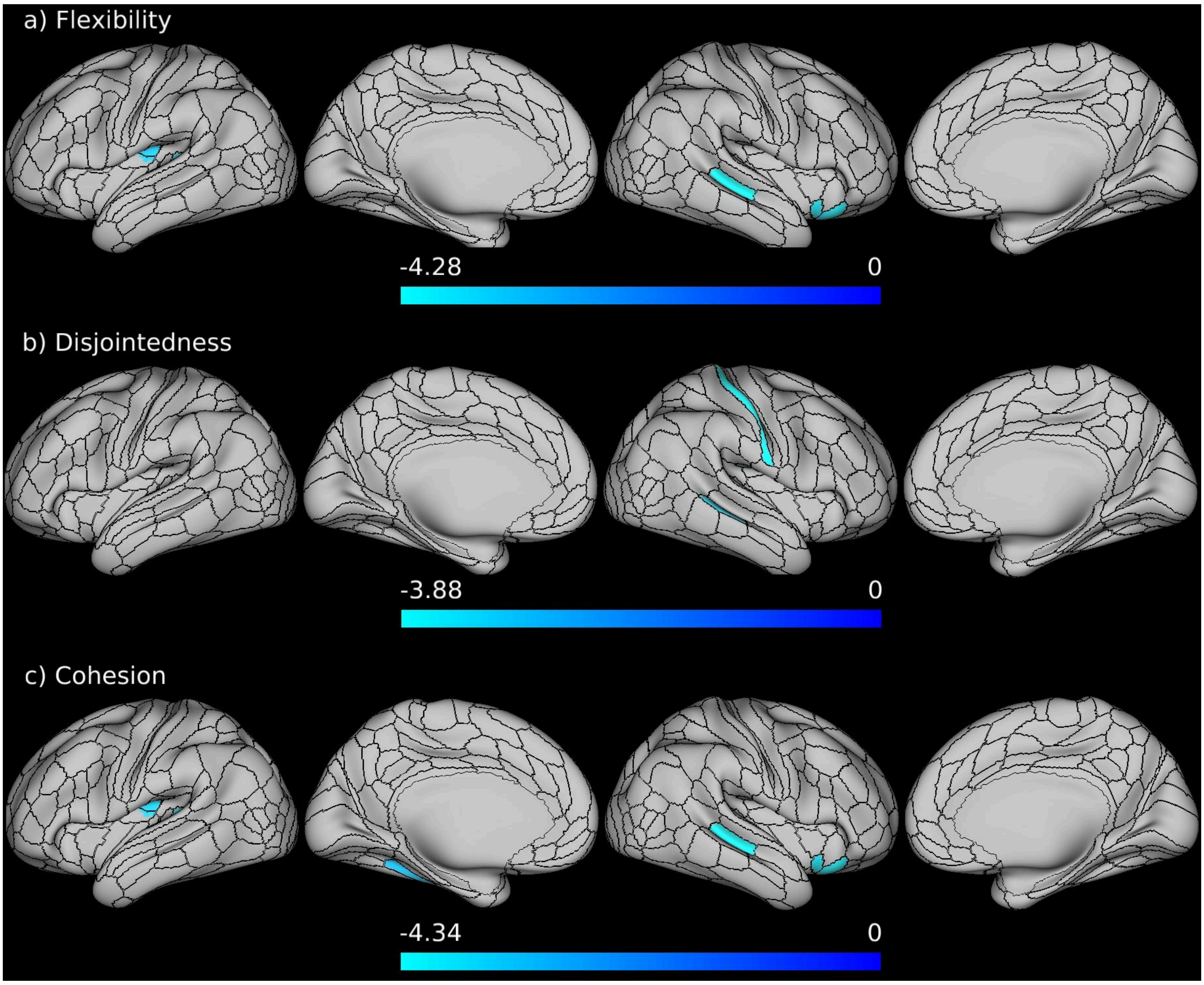

Supplementary Figure 16 Nodal dynamic network reconfiguration measures that were significantly associated with the MDS-UPDRS part 3 total score for sliding window length of 19 TRs in Parkinson's disease patients, using Glasser's atlas. Colorbars show t-values. There were no significant associations for promiscuity and subcortical regions.

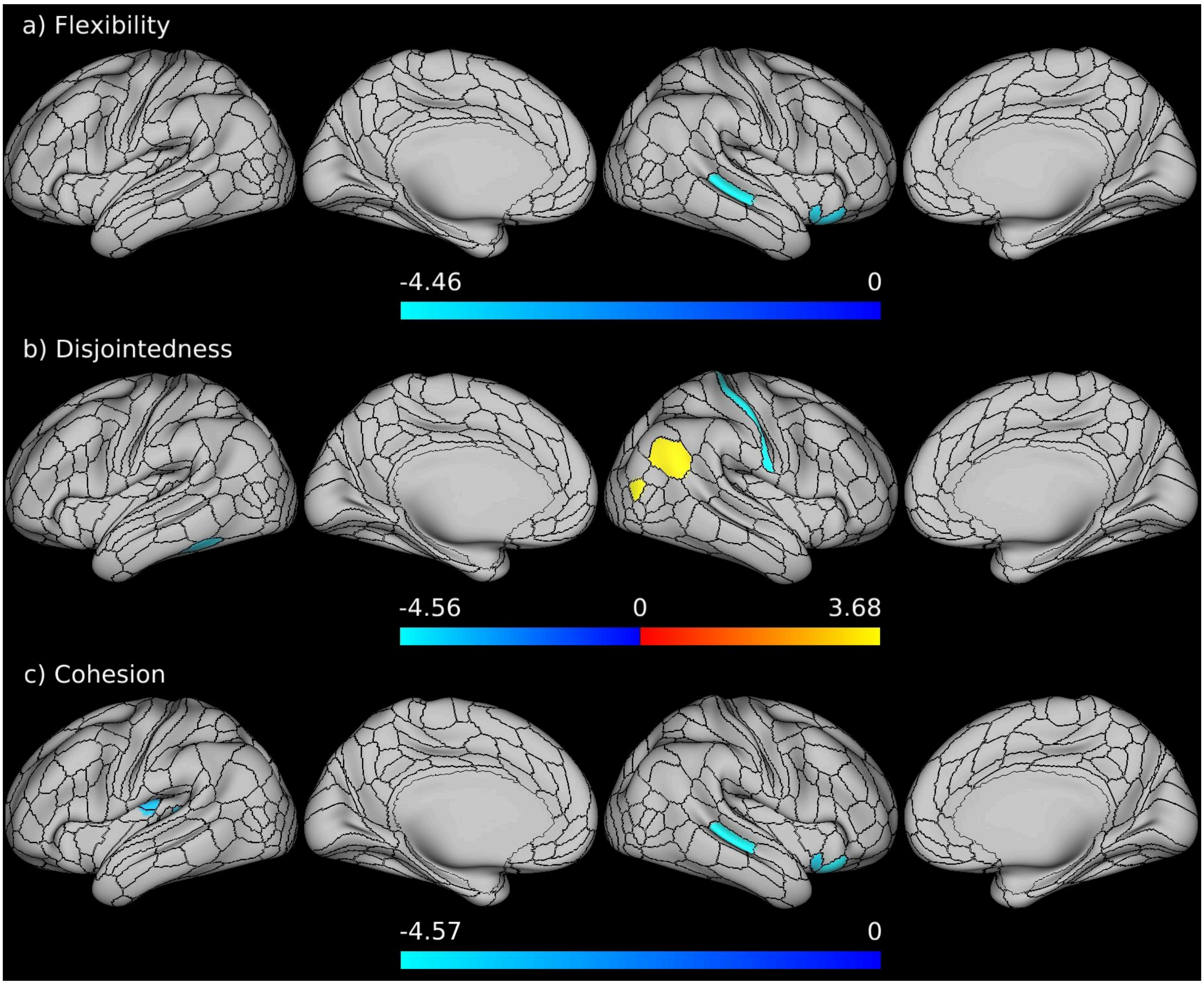

Supplementary Figure 17 Nodal dynamic network reconfiguration measures that were significantly associated with the MDS-UPDRS part 3 total score for sliding window length of 23 TRs in Parkinson's disease patients, using Glasser's atlas. Colorbars show t-values. There were no significant associations for promiscuity and subcortical regions.

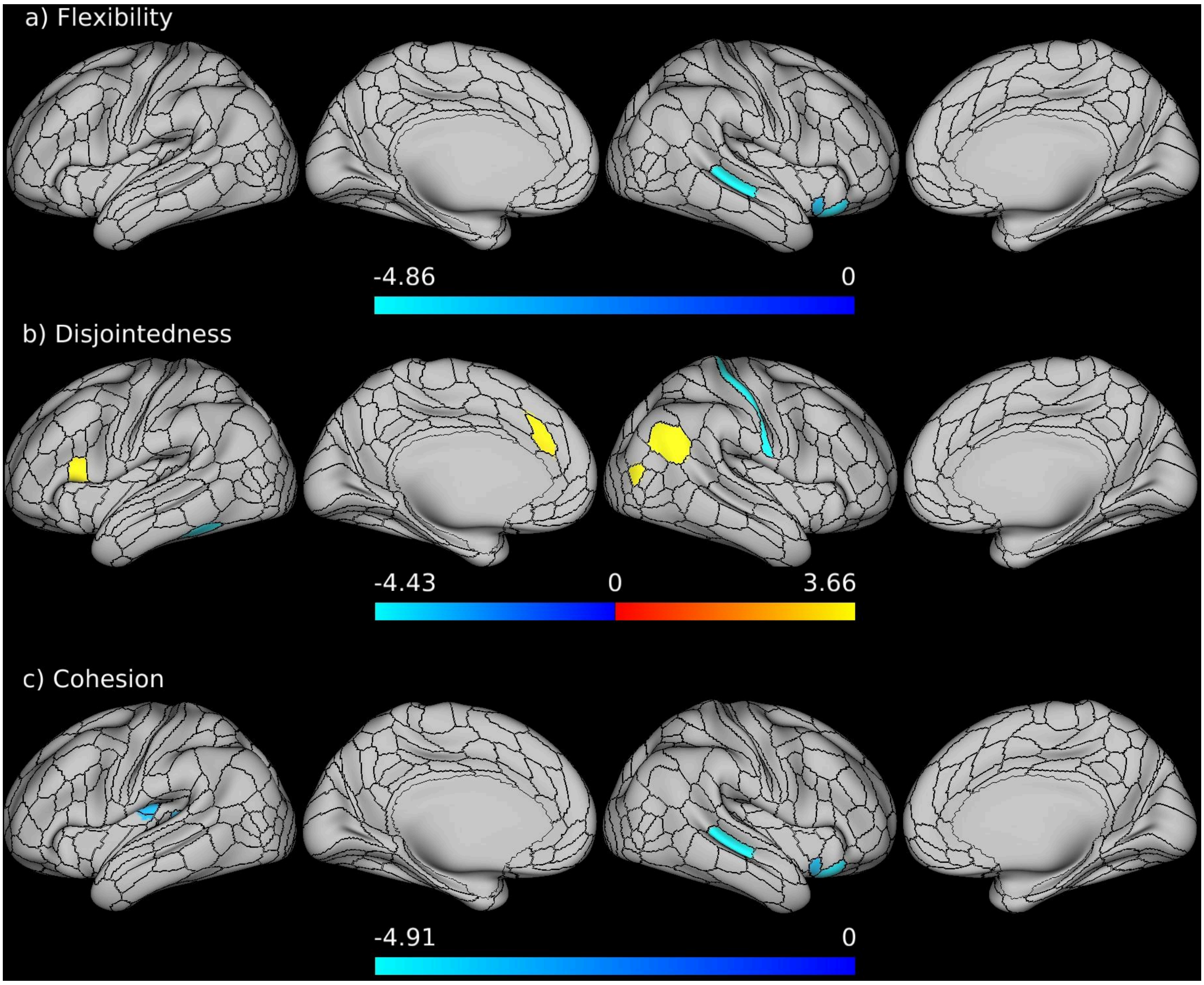

Supplementary References

1. Tustison NJ, Avants BB, Cook PA, et al. N4ITK: Improved N3 Bias Correction. *IEEE Trans Med Imaging*. 2010;29(6):1310-1320.

2. Avants BB, Epstein CL, Grossman M, Gee JC. Symmetric diffeomorphic image registration with cross-correlation: Evaluating automated labeling of elderly and neurodegenerative brain. *Med Image Anal*. 2008;12(1):26-41.

3. Zhang Y, Brady M, Smith S. Segmentation of brain MR images through a hidden Markov random field model and the expectation-maximization algorithm. *IEEE Trans Med Imaging*. 2001;20(1):45-57.

4. Dale AM, Fischl B, Sereno MI. Cortical Surface-Based Analysis: I. Segmentation and Surface Reconstruction. *NeuroImage*. 1999;9(2):179-194.

5. Klein A, Ghosh SS, Bao FS, et al. Mindboggling morphometry of human brains. *PLoS Comput Biol*. 2017;13(2):e1005350.

6. Evans AC, Janke AL, Collins DL, Baillet S. Brain templates and atlases. *NeuroImage*. 2012;62(2):911-922.

7. Cox RW, Hyde JS. Software tools for analysis and visualization of fMRI data. *NMR Biomed*. 1997;10(4-5):171-178.

8. Greve DN, Fischl B. Accurate and robust brain image alignment using boundary-based registration. *NeuroImage*. 2009;48(1):63-72.

9. Jenkinson M, Bannister P, Brady M, Smith S. Improved Optimization for the Robust and Accurate Linear Registration and Motion Correction of Brain Images. *NeuroImage*. 2002;17(2):825-841.

10. Power JD, Mitra A, Laumann TO, Snyder AZ, Schlaggar BL, Petersen SE. Methods to detect, characterize, and remove motion artifact in resting state fMRI. *NeuroImage*. 2014;84:320-341.

11. Behzadi Y, Restom K, Liau J, Liu TT. A component based noise correction method (CompCor) for BOLD and perfusion based fMRI. *NeuroImage*. 2007;37(1):90-101.

12. Satterthwaite TD, Elliott MA, Gerraty RT, et al. An improved framework for confound regression and filtering for control of motion artifact in the preprocessing of resting-state functional connectivity data. *NeuroImage*. 2013;64:240-256.

13. Lanczos C. Evaluation of Noisy Data. *J Soc Ind Appl Math Ser B Numer Anal*. 1964;1(1):76-85.

14. Abraham A, Pedregosa F, Eickenberg M, et al. Machine learning for neuroimaging with scikit-learn. *Front Neuroinform*. 2014;8. https://www.frontiersin.org/articles/10.3389/fninf.2014.00014

15. Mehta K, Salo T, Madison TJ, et al. XCP-D: A robust pipeline for the post-processing of fMRI data. *Imaging Neuroscience*. 2024;2:1-26.

16. Gorgolewski K, Burns C, Madison C, et al. Nipype: A Flexible, Lightweight and Extensible Neuroimaging Data Processing Framework in Python. *Front Neuroinform*. 2011;5. https://www.frontiersin.org/articles/10.3389/fninf.2011.00013

17. Parkes L, Fulcher B, Yücel M, Fornito A. An evaluation of the efficacy, reliability, and sensitivity of motion correction strategies for resting-state functional MRI. *Neuroimage*. 2018;171:415-436.

18. Lydon-Staley DM, Ciric R, Satterthwaite TD, Bassett DS. Evaluation of confound regression strategies for the mitigation of micromovement artifact in studies of dynamic resting-state functional connectivity and multilayer network modularity. *Netw Neurosci*. 2019;3(2):427-454.

19. Ciric R, Rosen AFG, Erus G, et al. Mitigating head motion artifact in functional connectivity MRI. *Nat Protoc*. 2018;13(12):2801-2826.

20. Schaefer A, Kong R, Gordon EM, et al. Local-Global Parcellation of the Human Cerebral Cortex from Intrinsic Functional Connectivity MRI. *Cereb Cortex*. 2017;28(9):3095-3114.

21. Tian Y, Margulies DS, Breakspear M, Zalesky A. Topographic organization of the human subcortex unveiled with functional connectivity gradients. *Nat Neurosci*. 2020;23(11):1421-1432.

22. Cox RW. AFNI: software for analysis and visualization of functional magnetic resonance neuroimages. *Comput Biomed Res*. 1996;29(3):162-173.

23. Ciric R, Thompson WH, Lorenz R, et al. TemplateFlow: FAIR-sharing of multi-scale, multi-species brain models. *Nat Methods*. 2022;19(12):1568-1571.

24. Hunter JD. Matplotlib: A 2D Graphics Environment. *Comput Sci Eng*. 2007;9(3):90-95.

25. Brett MA, Markiewicz CJA, Hanke MA, et al. *Nipy/nibabel: 5.0.1*. Zenodo; 2023. doi:10.5281/zenodo.7633628

26. Harris CR, Millman KJ, van der Walt SJ, et al. Array programming with NumPy. *Nature*. 2020;585(7825):357-362.

27. Yarkoni T, Markiewicz CJ, de la Vega A, et al. PyBIDS: Python tools for BIDS datasets. *J Open Source Softw*. 2019;4(40).

28. Virtanen P, Gommers R, Oliphant TE, et al. SciPy 1.0: fundamental algorithms for scientific computing in Python. *Nat Methods*. 2020;17(3):261-272.
